# The cost-effectiveness of antenatal and postnatal education and support interventions for women aimed at promoting breastfeeding in the UK

**DOI:** 10.1186/s12889-021-12446-5

**Published:** 2022-01-22

**Authors:** Ifigeneia Mavranezouli, Jo Varley-Campbell, Sarah Stockton, Jennifer Francis, Clare Macdonald, Sunita Sharma, Peter Fleming, Elizabeth Punter, Charlotte Barry, Maija Kallioinen, Nina Khazaezadeh, David Jewell

**Affiliations:** 1 Centre for Outcomes Research and Effectiveness, Research Department of Clinical, Educational & Health Psychology, University College London, 1-19 Torrington Place, London, WC1E 7HB UK; 2 National Guideline Alliance, Royal College of Obstetricians and Gynaecologists, London, UK; 3 Central Surgery, Oadby, Leicestershire, UK; 4 Institute of Applied Health Research, University of Birmingham, Birmingham, UK; 5 Chelsea and Westminster Hospital NHS Foundation Trust, London, UK; 6 Centre for Academic Child Health, Bristol Medical School, University of Bristol, Bristol, UK; 7 The Dudley group NHS foundation Trust, Dudley, West Midlands UK; 8 Midlands Maternity and Perinatal Mental Health Clinical Network, NHS England and NHS Improvement, London, UK; 9 Guy’s & St. Thomas NHS Foundation Trust, London, UK; 10 Chair of the committee responsible for the NICE guideline on postnatal care and General Practitioner, Bristol, UK

**Keywords:** Breastfeeding interventions, Decision-analytic modelling, Economic evaluation, Cost-effectiveness

## Abstract

**Background:**

Breastfeeding is associated with health benefits to mothers and babies and cost-savings to the health service. Breastfeeding rates in the UK are low for various reasons including cultural barriers, inadequate support to initiate and sustain breastfeeding, lack of information, or choice not to breastfeed. Education and support interventions have been developed aiming at promoting breastfeeding rates. The objective of this study was to assess the cost-effectiveness of such interventions for women, initiated antenatally or in the first 8 weeks postnatally, aiming at improving breastfeeding rates, in the UK.

**Methods:**

A decision-analytic model was constructed to compare costs and quality-adjusted life-years (QALYs) of a breastfeeding intervention from the perspective of health and personal social services in England. Data on intervention effectiveness and the benefits of breastfeeding were derived from systematic reviews. Other model input parameters were obtained from published sources, supplemented by expert opinion.

**Results:**

The incremental cost-effectiveness ratio (ICER) of the modelled intervention added on standard care versus standard care was £51,946/QALY, suggesting that the intervention is not cost-effective under National Institute for Health and Care Excellence (NICE) criteria in England. Sensitivity analysis suggested that the cost-effectiveness of the intervention improved as its effectiveness increased and intervention cost decreased. At the base-case effect (increase in breastfeeding rates 16–26 weeks after birth by 19%), the intervention was cost-effective (<£20,000/QALY) if its cost per woman receiving the intervention became ≈£40–£45. At the base-case cost (£84), the intervention was cost-effective if it increased breastfeeding rates by at least 35–40%.

**Conclusions:**

Available breastfeeding interventions do not appear to be cost-effective under NICE criteria in England. Future breastfeeding interventions need to have higher effectiveness or lower cost compared with currently available interventions in order to become cost-effective. Public health and other societal interventions that protect, promote and support breastfeeding may be key in improving breastfeeding rates in the UK.

**Supplementary Information:**

The online version contains supplementary material available at 10.1186/s12889-021-12446-5.

## Background

Breastfeeding is associated with important health benefits to both babies and breastfeeding women [1]. Nevertheless, global rates of breastfeeding are well below targets set by the World Health Assembly [2]. The UK has some of the lowest breastfeeding rates in the world with reported barriers to breastfeeding including a lack of access to support services in the community and at work, cultural barriers and misinformation on the benefits and practicalities of infant feeding [3]. Various interventions have been developed over the years, aimed at promoting initiation and/or maintenance of breastfeeding [4–6]. Existing evidence on the cost-effectiveness of such interventions is limited and has not considered the long-term benefits to women and their babies and related cost-savings associated with breastfeeding [7–11]. The objective of this study was therefore to examine the long-term cost-effectiveness of interventions for women, initiated antenatally or in the first 8 weeks after birth, aimed at promoting initiation and/or maintenance of breastfeeding from the perspective of the National Health Service (NHS) and Personal Social Services (PSS) in England, using decision-analytic economic modelling.

The analysis presented here is part of the work that informed the updating of national guidance on postnatal care in England, published by the National Institute for Health and Care Excellence (NICE) [12]. The guideline was developed by a guideline committee, an independent multi-disciplinary group of clinical academics, health professionals and service users and carers with expertise and experience in the area of postnatal care. The committee contributed to the development of the economic model by providing advice on issues relating to the provision of breastfeeding interventions and associated benefits and resource use savings. They also contributed to the economic model structure and advised on model inputs in areas where there was paucity of evidence.

## Methods

### Population

The study population comprised women who are pregnant or have given birth to healthy babies at term, and their babies. The women’s age at the start of the economic model was 30 years, as this is the mean age of women giving birth in England and Wales [13]. The starting age of the cohort was needed in order to model lifetime benefits to women associated with breastfeeding. In sensitivity analyses we varied the age of women when receiving the intervention from 25 to 35 years to explore whether this has an impact on the results. Women could have had a singleton, twin or higher order live birth. In accordance with national epidemiological data, the mean number of babies per delivery of liveborns was 1.016 [14].

Women received the intervention at one point in time, and, in the base-case analysis, the intervention’s effect was applied only on that birth and not on future ones. In sensitivity analysis we maximised the impact of the intervention by assuming that all women received the intervention when they had their first baby and that the effect of the intervention on breastfeeding rates would be retained in all subsequent births, using the total fertility rate of 1.70 births/woman in England [14].

### Intervention

The characteristics of the breastfeeding intervention regarding effectiveness and resource use (number of sessions, format, people delivering the intervention, etc.) were informed by the findings of a systematic review and meta-regression of 62 randomised controlled trials (RCTs) undertaken to inform the NICE guideline [15]; resource use characteristics were further supplemented by the committee’s expert opinion to reflect routine practice in the UK. The focus of the analysis was an intervention for women that comprised education, advice or support from a peer or professional, provided postnatally and initiated antenatally or within the first eight weeks after birth, as the majority of clinical evidence was available for this type of intervention. Broader public health interventions that aim to promote breastfeeding were beyond the guideline scope. In accordance with available evidence, the intervention was assumed to be provided in addition to standard care; the comparator of the analysis was standard care alone. The definition of standard care varied widely across RCTs included in the systematic review and meta-regression that informed the economic analysis. Standard care ranged from no intervention, through written materials and peer breastfeeding support, to availability of breastfeeding educational programmes of variable intensity in-hospital or in the community. In England, standard care is also variable and may include provision of written material, antenatal breastfeeding educational programmes, and postnatal breastfeeding support groups run by peers and/or health professionals; in most settings breastfeeding information and support is provided by midwives and health visitors as part of routine postnatal care visits.

In order to specify the intervention and identify its effective components, effectiveness data on ‘any breastfeeding between 16 and 26 weeks after birth’, obtained from the systematic review and meta-regression, were inspected (Table 1). Data on ‘any breastfeeding’, rather than ‘exclusive breastfeeding’ were selected because most of the available data on the protective effects of breastfeeding that informed the economic model were relevant to ‘any’ breastfeeding (more versus less, longer versus shorter duration, any versus none, etc.) rather than ‘exclusive’ breastfeeding; moreover, the period of 16–26 weeks after birth was chosen to ensure that breastfeeding was established and therefore could have an impact on longer-term mother and baby outcomes, and during this period no efficacy data on exclusive breastfeeding were available. The components of the modelled intervention were specified by looking at the intervention characteristics that demonstrated a statistically significant effect (risk ratio, RR) versus standard care. Face-to-face interventions, delivered either individually or in group format, and also interventions delivered remotely, appeared to be effective compared with standard care. However, the group intervention effect was by far the largest observed in our meta-regression and only based on a single small study (*N* = 100; in comparison, the total number of participants across studies included in that meta-regression was *N* = 14,229); hence the committee did not consider the group intervention effect further when specifying the characteristics of the modelled intervention. Interventions comprising 4–8 contacts appeared to have the greatest effect. Interventions seemed to be effective if they were delivered at home or in a mixed home and healthcare setting.

**Table 1:** Effectiveness of interventions aimed at promoting breastfeeding – results of meta-regression for ‘any breastfeeding 16 to 26 weeks after birth’ [15]

Comparisons – every component vs standard care	Risk Ratio	Lower 95% CI	Upper 95% CI
**Any intervention**	**1.08**	**1.03**	**1.13**
*How*			
**Face-to-face individual**	**1.07**	**1.01**	**1.14**
**Face-to-face group**	**1.95**	**1.45**	**2.27**
**Remote**	**1.15**	**1.05**	**1.26**
Self-help	1.06	0.74	1.40
*Number of Contacts*			
0	1.18	0.96	1.39
1	1.05	0.95	1.14
2–3	1.07	0.97	1.17
**4–8**	**1.19**	**1.10**	**1.30**
**9**	**1.13**	**1.00**	**1.26**
*Duration of intervention*			
Less than 8 weeks	1.04	0.97	1.10
**More than 8 weeks**	**1.20**	**1.11**	**1.29**
*Where delivered*			
**Home**	**1.12**	**1.05**	**1.19**
Healthcare setting	1.06	0.96	1.17
**Mixed home/healthcare setting**	**1.16**	**1.03**	**1.30**

Comparisons with statistically significant effects at the *p* ≤ 0.05 level have been highlighted in bold. *CI* confidence interval

#### Effectiveness of the modelled intervention

The economic analysis utilised the effect on any breastfeeding at 16–26 weeks after birth for “4-8 contacts vs standard care” [mean RR 1.19, 95% confidence intervals (CI) 1.10 to 1.30] [15]. Sensitivity analysis explored the impact of changes in the mean effect (range of RR from 1.05 to 2.00) on the cost-effectiveness of the intervention.

#### Modelled intervention cost

The intervention cost was estimated using national unit costs [16] and expert advice, assuming that the intervention consisted of 6 contacts, i.e. the average of 4–8 contacts which was the most effective number of contacts identified in the meta-regression [15]. Based on the committee’s advice on patterns of routine practice in England, we made the following assumptions regarding the delivery of the intervention: four contacts comprised 30-min individual face-to-face sessions, and two further contacts comprised 45-min group face-to-face sessions delivered to groups of 6 women. The first two individual sessions were provided by a health professional on a NHS England Agenda for Change Band 5 salary. The remaining two individual and the two group sessions were provided by a volunteer trained peer supporter. The total estimated intervention cost was £84 (Table 2). The intervention was offered in addition to standard care, and therefore the description and cost of standard care was omitted from both arms of the model. Sensitivity analysis explored the impact of changes in the intervention cost (range from £20 to £100) on the cost-effectiveness of the intervention.

**Table 2:** Cost of intervention aimed at promoting breastfeeding

Cost element	Unit cost	Cost per woman
2 individual face-to-face sessions lasting 30 min each (total 60 min), provided by a health professional in NHS England Agenda for Change (AfC) Band 5 (nursing, midwifery and health visiting staff).	£59 per patient-related hour^a^	**£59**
2 individual face-to-face sessions lasting 30 min each (total 60 min), delivered by a volunteer trained peer supporter.	£20 per patient-related hour^b^	**£20**
2 group face-to-face sessions delivered to groups of 6 women, lasting 45 min each (total 90 min / 6 women = 15 min per woman), delivered by a volunteer trained peer supporter.	£20 per patient-related hour^b^	**£5**
**TOTAL COST PER WOMAN**		**£84**

^a^ [16]. Mean annual basic pay £26,231. Unit cost includes salary, salary on-costs and overheads; actual working time and the ratio of direct time (direct care) to indirect time (care planning, assessment and co-ordination, travelling, administrative tasks and other duties) taken into account. Travel expenses not included due to lack of relevant data

^b^ Expert advice. Unit cost includes training, supervision, co-ordination and travel, but not childcare

### Baseline probability of breastfeeding

Current breastfeeding rates under standard care for the period of 16 weeks (4 months) to 26 weeks (6 months) after birth were obtained from national statistics.

For baby outcomes, baseline rates of any breastfeeding at 4 months after birth were used, as breastfeeding is established by this time point, leading to health benefits, and evidence suggests that the protective effect of breastfeeding is retained after this point even after breastfeeding stops [1]. For breast cancer in mothers, baseline rates of any breastfeeding at 6 months after birth were used, as evidence suggests that the effect of breastfeeding on the incidence of breast cancer may be significant from 6 months of breastfeeding onwards [17, 18].

The most recent (2019) data on any breastfeeding were only available for 6–8 weeks after birth [19]. The most recent rates of any breastfeeding at 4 and 6 months after birth in England were available for the year 2010 [20]. Nevertheless, it was possible to estimate the rates of any breastfeeding at 4 and 6 months after birth for 2019, using the 2019 data on the prevalence of any breastfeeding at 6–8 weeks and the instant rate of reduction in any breastfeeding between 6 weeks and 4 months (16 weeks) and between 4 months and 6 months (26 weeks) as calculated from the available 2010 data. In order to estimate the rates of breastfeeding at 4 and 6 months after birth in 2019 using the available data, we assumed an exponential decrease in breastfeeding rates, which was more rapid between 6 weeks and 4 months compared with the period between 4 and 6 months, according to available data. This assumption was necessary due to lack of more detailed data that would allow us to determine the rate of decrease in breastfeeding rates more accurately over time. The actual and estimated rates of any breastfeeding at different time points following birth for the years 2010 and 2019 are shown in Table 3.

**Table 3:** Prevalence of any breastfeeding in England at different time points after birth

Time point	Prevalence of any breastfeeding	
	2010 [20]	2019 [19]
Birth	83%	
6–8 weeks after birth	57% [6 weeks]	53% [6–8 weeks, cases with known status only]
4 months after birth	44%	42% [estimated]^a^
6 months after birth	36%	34% [estimated]^a^

^a^ estimated using the 2019 figure for the prevalence of any breastfeeding at 6–8 weeks and the instant rate of reduction in any breastfeeding between 6 weeks and 4 months, and between 4 months and 6 months, as calculated from the 2010 data (assuming exponential decrease in breastfeeding rates)

### Overview of costs and benefits considered in the analysis

Costs consisted of the intervention cost and costs associated with breastfeeding outcomes that are incurred in community, primary or secondary healthcare or personal social service settings. Costs to parents relating to either formula feeding (milk powder, bottles, sterilising equipment) or breastfeeding (breast pumps, bottles, sterilising equipment, nursing bras, nipple pads) were not considered as these were outside the NHS/PSS perspective of the analysis. The cost year was 2018. The primary outcome measure was the QALY. Other secondary outcome measures were determined by, and are specific to, the clinical conditions considered in the analysis, and are described under ‘Model structure’ together with each relevant model component.

### Selection of clinical conditions associated with breastfeeding for inclusion in modelling

A systematic review of studies that modelled long-term clinical benefits to mothers and babies (and/or related cost-savings to health and personal social services) associated with breastfeeding was undertaken in order to identify data on long-term clinical outcomes associated with breastfeeding, as well as relevant epidemiological and resource use data that could be adopted or adapted to inform our economic analysis. Details of the review are provided in the guideline evidence report [15]. Included studies and studies excluded after full text was obtained are provided in Supplementary File 1.

The review identified two studies of high quality and directly relevant to our study’s objective, that is, the modelling of long-term outcomes and cost-savings associated with breastfeeding [1, 18]. Renfrew et al. [18] developed an economic model to estimate long-term benefits to mothers and babies and cost-savings to the UK healthcare system associated with breastfeeding. The study, which was commissioned by UNICEF UK, was informed by high quality systematic reviews regarding the benefits of breastfeeding to mothers and babies. Victora et al. [1] examined the association between breastfeeding and clinical outcomes to mothers and babies based on the results of 28 systematic reviews and meta-analyses, 22 of which were commissioned by the World Health Organization (WHO). These two studies alone reported all data on the association between breastfeeding and mother and baby outcomes that had informed the remaining modelling studies. Only one modelling study [21] used data on the association between breastfeeding and breast cancer that had not been already reported by either Renfrew et al. [18] or Victora et al. [1], which were obtained from a more recent meta-analysis published in 2017 [22].

Based on the review findings, we decided to use the analysis undertaken by Renfrew et al. [18] as the starting point for our analysis, regarding the selection and modelling of clinical benefits (and related NHS/PSS cost-savings) associated with breastfeeding, and update the data on the association between breastfeeding and clinical outcomes using, where available, more up-to-date evidence reported in Victora et al. [1]. The Renfrew et al. study [18] was selected for this purpose because it considered a range of clinical conditions, was of high quality and utilised UK-specific epidemiological and resource use data. Based on the evidence from these two studies, we selected clinical conditions associated with breastfeeding for inclusion in our analysis, also taking into account feasibility issues and the expected magnitude of clinical benefits and cost-savings per person associated with a change in breastfeeding rates. The evidence we reviewed and the considerations that led to selection of clinical conditions are shown in Supplementary File 2. The clinical conditions associated with breastfeeding that were selected for inclusion in our economic analysis are shown in Table 4.

**Table 4:** Clinical conditions associated with breastfeeding that were considered in the economic analysis

Clinical conditions in babies	Clinical conditions in mothers
• Gastrointestinal infection (GI) [diarrhoea attributable to infection] • Respiratory tract infection (RTI) • Acute otitis media (AOM) • Mortality due to infectious diseases • Mortality due to sudden infant death syndrome (SIDS)	• Breast cancer

### Model structure

A hybrid decision-analytic model was constructed using Microsoft Office Excel 2013 to estimate total NHS/PSS costs and benefits to mothers and babies associated with the provision of a breastfeeding intervention. The structure of the model, which aimed to simulate the course of a number of clinical conditions whose incidence is associated with breastfeeding, was driven by patterns of clinical practice in the UK and the availability of relevant clinical data.

According to the model structure, hypothetical cohorts of women who are pregnant or have given birth to healthy babies at term were either initiated on a breastfeeding intervention in addition to standard care, or received standard care only. Following care, women either breastfed or did not breastfeed their babies at 16–26 weeks after birth. Women and their babies were subsequently followed up for a period of time that ranged from one year after birth to lifetime, depending on the clinical condition assessed, to estimate their outcomes and associated costs resulting from their breastfeeding status at 16–26 weeks after birth.

The first part of the economic model, which assessed the impact of the breastfeeding intervention on breastfeeding rates at 16–26 weeks after birth (effectiveness of intervention), took the form of a decision tree. This was followed by separate models on each of the clinical conditions considered for mothers and babies, which took the form of either decision trees or Markov models, as appropriate for the condition examined.

The models on gastrointestinal infection (GI), respiratory tract infection (RTI) and acute otitis media (AOM) in babies took the form of a simple decision tree, where babies either developed one of the infections or not, following the structure in the Renfrew et al. model [18]. Those who developed an infection were treated by GPs (with each infection assumed to correspond to one GP contact), with a number of babies developing GI and RTI being hospitalised for further treatment. The time horizon of those models was one year. The outcome measures were the number of cases of GI, lower RTI and AOM as well as the number of hospitalisations due to GI and RTI in babies aged up to one year that were prevented by breastfeeding. These were secondary outcomes in the analysis.

One model was developed for mortality due to infectious diseases or sudden infant death syndrome (SIDS) in babies. Babies who did not die due to infectious diseases or SIDS over their first year of life owing to the protective effect of breastfeeding entered a very simple, two-state Markov model, with a one-year cycle, that considered the states of ‘alive’ and ‘dead’ over the babies’ lifetimes. This was a new model as Renfrew et al. [18] did not consider mortality due to infectious diseases in their economic modelling, and assessed cost-savings and outcomes associated with SIDS in a narrative synthesis. The outcome measures of this model component were the number of QALYs gained over saved babies’ lifetime (primary outcome) and the number of deaths due to infectious diseases or SIDS prevented in babies aged up to one year (secondary outcome).

One three-state Markov model was developed to assess costs and outcomes for women at risk for breast cancer starting from 30 years of age and over their lifetime. The model, which considered the protective effect of breastfeeding on the risk of breast cancer over women’s lifetime, included the states of ‘no breast cancer’, ‘breast cancer’ and ‘death’; the model cycle was one year and a half-cycle correction was applied. This model had the same overall structure as the Renfrew et al. study [18], but adopted a different approach and considered more parameters associated with the risk of breast cancer in parous women, employed different assumptions to model the course of disease (in particular mortality), and utilised different epidemiological, utility and cost data on breast cancer.

Breast cancer in women who survived was assumed to last 10 years, after which women who survived re-entered the ‘no breast cancer’ state and were at risk of developing a new breast cancer. The state of ‘breast cancer’ consisted of 10 tunnel states, one for each year of breast cancer, so that the time women spent with breast cancer could be estimated and a breast cancer’s duration-dependent mortality, as well as time-dependent costs and utilities associated with breast cancer, could be applied. The outcome measures of this model component were the number of QALYs (primary outcome) and the number of new cases of breast cancer prevented over lifetime (secondary outcome).

**Fig. 1:**
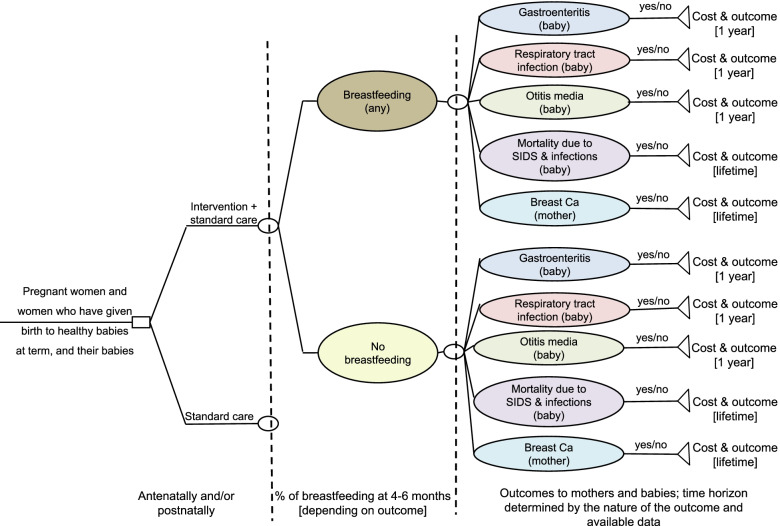
Schematic structure of the economic model assessing the cost-effectiveness of an intervention for women aiming at starting and maintaining breastfeeding

**Fig. 2:**
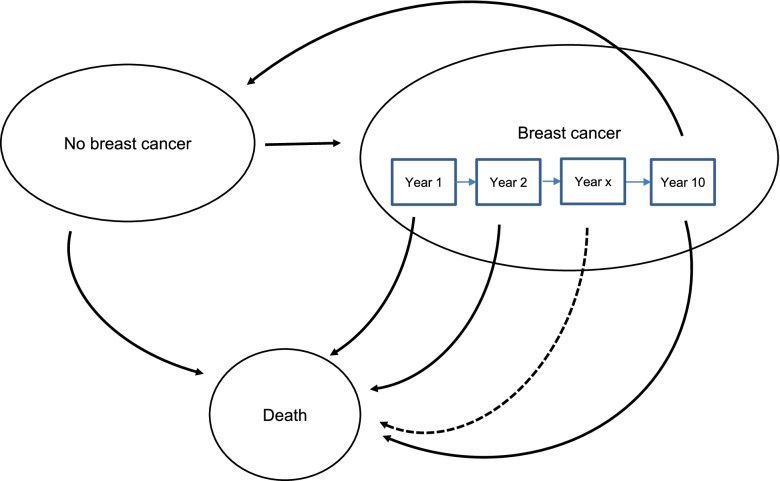
Schematic structure of the economic model component on mothers’ breast cancer

The overall structure of the economic model assessing the cost-effectiveness of an intervention for starting and maintaining breastfeeding is shown in Fig. 1. Figure 2 shows the economic model component on mothers’ breast cancer.

Our model was built on the evidence that provision of a breastfeeding intervention increases breastfeeding rates; the protective effect of increased breastfeeding on various clinical conditions was subsequently applied onto the current (baseline) incidence of each clinical condition under standard care, to estimate the reduction in incidence following intervention. The model took into account the fact that the current (baseline) incidence of each clinical condition reflects the current mix of mothers and babies who breastfeed/are breastfed and those who do not breastfeed/are not breastfed, respectively (i.e. all mothers and their healthy babies born at term under standard care).

To estimate the incidence of each clinical condition under current standard practice in mothers that breastfeed or babies that are breastfed (BF) and those that do not breastfeed / are not breastfed, respectively (nonBF), the following formulae were used [23]:

$$Incidence\ in\ nonBF=\frac{Overall\ in cidence}{Current\ BF\ rate\ x\ RR+1- current\ BF\ rate}$$

and

$$Incidence\ in\ BF= Incidence\ in\ nonBF\ x\ RR$$

where ‘overall incidence’ is the incidence of the clinical condition in the overall population of mothers or babies, and RR the risk ratio expressing the protective effect of breastfeeding on the clinical condition assessed. These formulae utilise RR to express the protective effect of breastfeeding. On the other hand, the protective effect of breastfeeding has been expressed as odds ratio (OR) for some clinical conditions. However, when the incidence of an event at baseline is rare (< 10%), then OR approximates RR and the formulae can produce accurate results using OR instead of RR [24].

### Data on the association of breastfeeding with the clinical conditions considered in the economic analysis

We utilised data from Victora et al. [1] for the following outcomes:

i) incidence of diarrhoea (reflecting GI) in babies and children aged 6 months - 5 years (RR of more versus less breastfeeding: 0.46, 95% CI 0.28 to 0.78)
ii) hospitalisation due to diarrhoea (reflecting GI) in babies and children aged ≤5 years (RR of more versus less breastfeeding: 0.28, 95% CI 0.16 to 0.50)
iii) incidence or prevalence of lower RTI in babies and children aged < 2 years (RR of more versus less breastfeeding: 0.68, 95% CI 0.60 to 0.77)
iv) hospitalisation due to RTI (both lower and upper, according to available evidence) in babies and children aged < 2 years (RR of more versus less breastfeeding: 0.43, 95% CI 0.33 to 0.55)
v) incidence of AOM in babies and children aged < 2 years (OR of more versus less breastfeeding: 0.67, 95% CI 0.62 to 0.72)
vi) mortality due to infectious diseases in babies and children aged 6–23 months (OR of any versus no breastfeeding: 0.48, 95% CI 0.38 to 0.60)
vii) mortality due to SIDS in babies and children aged ≥2 months (OR of any versus no breastfeeding: 0.38, 95% CI 0.27 to 0.54).

Updated evidence suggests a protective effect of breastfeeding on the incidence of and hospitalisations due to GI in babies over the first 5 years of their life, although this effect appears to be stronger in younger ages. Moreover, breastfeeding is associated with a reduction in the incidence of lower RTI and AOM and hospitalisation due to RTI in babies over the first 2 years of their life. Nevertheless, we conservatively estimated costs and outcomes associated with incidence of GI, lower RTI and AOM and hospitalisation due to GI and RTI in babies up to their first year of life, to retain consistency with the Renfrew et al. model [18] and because relevant epidemiological data were available for babies up to one year of age.

Data on the protective effect of breastfeeding on the incidence of breast cancer were obtained from a published meta-analysis [22], which pooled data from 25 studies on parous women and adjusted for several confounders such as age, parity, age at first pregnancy and family history of breast cancer. The standardised RR for breast cancer in parous women for any versus no breastfeeding for 6 months was used (0.86, 95% CI 0.82 to 0.91).

### Baseline risks and mortality

For the baseline incidence of GI, lower RTI and AOM in babies aged 0–1 years in England we used the number of GP consultations for this age group for the clinical diagnoses of (a) diarrhoea, intestinal infectious diseases, non-infective enteritis, and colitis (4682 per 100,000) to reflect consultations for GI; (b) lower RTI (23,433 per 100,000); and (c) AOM (13,556 per 100,000). These data were reported in Renfrew et al. [18], derived from the Royal College of General Practitioners (RCGP) database.

The baseline rates of hospital admissions due to GI and RTI over the first year of life (15.3 and 115.4 per 1000 live births, respectively) were estimated using data on admissions for infectious intestinal diseases (reflecting GI) and RTI in babies aged 0–1 years in England [25], divided by the population aged 0–1 years in England [26].

The baseline mortality due to infectious diseases (12 per 100,000) and SIDS (25 per 100,000) in babies aged 0–1 years was estimated by dividing the number of deaths due to infectious diseases and SIDS in babies aged 0–1 years with the number of live births, using infant mortality data in England and Wales [27].

To estimate the annual mortality of babies who did not die from infectious diseases or SIDS owing to the protective effect of breastfeeding we used the proportion of males/females aged one year in England [26] and age- and gender-specific mortality over lifetime [28].

The baseline incidence of breast cancer in parous women was estimated using:

- The age-specific incidence of breast cancer in women in the general population, i.e. a mixture of parous and nulliparous women [29] (see Supplementary File 3).
- The percentage of nulliparous women in the population of women aged 30 years and over. This was 48% at 30 years of age; 27% at 35 years of age; 19% at 40 years of age; and 18% at 45 years of age and above [30].
- The mean number of children per parous woman aged ≥30 years (including previous births), which was approximately 2, starting from 1.90 at 30 years of age and reaching 2.23 at 45 years of age [30]. This parameter was used to estimate the incidence of breast cancer in parous women, as parity reduces the incidence of breast cancer and the reduction depends on the number of children per woman.
- The protective effect of parity on breast cancer, expressed as an OR of incidence of breast cancer in parous women with 2 live births versus non-parous women (0.84, 95% CI 0.80 to 0.89) [31]. Parous women with 2 live births were selected as the relevant sub-population of parous women, as the mean number of children of parous women aged ≥30 years (which is the study population) is 2, as reported above.

For every year in the model, the incidence of breast cancer in parous and nulliparous women was estimated using the formulae described earlier [23], using the overall age-specific incidence of breast cancer in women in the general population, the percentage of nulliparous women amongst women in the general population, and the protective effect of parity on breast cancer. The same formulae were used to estimate the incidence of breast cancer under current standard practice in women aged ≥30 years who breastfed and those who did not, amongst parous women.

Mortality in women without breast cancer was derived from age-specific mortality data for women in the general population [28]. For women with breast cancer, mortality in every model cycle was estimated using age-specific data on mortality of women in the general population [28], age-specific data on mortality due to breast cancer in women in the general population [32] (see Supplementary File 3), and age-adjusted net survival data for women with breast cancer over 1–10 years after diagnosis [33]. Using these data and a number of assumptions, it was possible to estimate the age- and breast cancer’s duration-specific mortality in women with breast cancer, depending on the number of years after diagnosis (that is, number of years lived with breast cancer). Details on the mortality data and the assumptions made in order to estimate mortality of women with and without breast cancer are reported in Supplementary File 3.

### Utility data

To estimate total QALYs over lifetime for babies who did not die from infectious diseases or SIDS owing to the protective effect of breastfeeding, as well as for women without breast cancer, age- and gender-specific EQ-5D-derived utility values for the UK population were used [34] (Table 5).

**Table 5:** Utility values of the general UK population - EQ-5D ratings [34]

Age (years)	Utility mean (standard error)	
	Males	Females
Under 25	0.94 (0.01)	0.94 (0.01)
25 to 34	0.93 (0.01)	0.93 (0.01)
35 to 44	0.91 (0.01)	0.91 (0.01)
45 to 54	0.84 (0.02)	0.85 (0.01)
55 to 64	0.78 (0.02)	0.81 (0.02)
65 to 74	0.78 (0.02)	0.78 (0.02)
75+	0.75 (0.03)	0.71 (0.02)

Utility values for women with breast cancer were estimated based on data reported in a systematic review and meta-analysis of utility values for breast cancer [35], after taking into account the proportion of women with de novo stage IV (metastatic) disease among prevalent cases of women with metastatic breast cancer [36], and the percentage of women with breast cancer that have metastases at diagnosis [37]. Details on the utility data reported in the study and further assumptions made for estimation of QALYs are provided in Supplementary File 3.

### Cost data utilised in the model

National sources were used to obtain the unit cost of a GP visit (£37) [16], as well as the cost of hospitalisation for GI (£756 per admitted child) and for RTI (£1094 per admitted child) [38]. The cost of death due to an infectious disease or SIDS per baby (£204) was also derived from national data [38] under the code VB99Z ‘Emergency medicine, patient dead on arrival’. This was the only cost considered for mortality in babies in the base-case analysis.

Babies dying from an infectious disease are likely to have incurred further healthcare costs, some of which may have already been considered under other modelled clinical conditions and were therefore not considered in this part of the model. Post-mortem examination costs may also be attached to the death of a baby. Thus, a sensitivity analysis explored the impact on the results of adding a post-mortem examination cost of £8000 (paediatric coronial case plus forensic examination) [39] to each baby’s death due to an infectious disease or SIDS. There are also considerable intangible emotional costs to parents following the death of a baby, which were not possible to include in the analysis.

Healthcare costs incurred by women with breast cancer and those without were obtained from a study on 359,771 women with breast cancer in England [40]. Details on the cost data reported in that study and the assumptions used in order to utilise available data in our model are provided in Supplementary File 3.

### Discounting

Where costs and/or outcomes were measured over a period longer than one year, they were discounted at an annual rate of 3.5% as recommended by NICE [41]. An annual discount rate of 1.5% was tested in sensitivity analysis, which is suggested by NICE for public health interventions [41].

### Analysis

To account for the uncertainty around input parameter point estimates, a probabilistic analysis was undertaken, in which input parameters were assigned probabilistic distributions [42]. Subsequently, 10,000 iterations were performed, each drawing random values out of the distributions fitted onto the model input parameters. Mean costs and outcomes for each strategy were calculated by averaging across the 10,000 iterations. The incremental cost-effectiveness ratio (ICER) was calculated using the formula:

$$\mathrm{ICER}=\Delta \mathrm{C}/\Delta \mathrm{E}$$

where ΔC is the difference in total costs between two treatment options considered and ΔE the difference in their effectiveness (QALYs). The ICER expresses the extra cost per extra unit of benefit (QALY) associated with one treatment option relative to its comparator. If an option has an ICER of up to £20,000–£30,000/QALY relative to its comparator (NICE lower and upper cost-effectiveness threshold, respectively) then the intervention is considered to be cost-effective [43].

Table 6 shows the deterministic values and probability distributions of all model input parameters and the methods employed to define their range. Deterministic values were used in a two-way sensitivity analysis, in which we changed concurrently the mean effect (RR) and cost of the intervention, to explore the impact of changes on the cost-effectiveness results. The ranges tested were from 1.05 to 2.00 for the intervention effect; and from £20 to £100 for the intervention cost.

**Table 6:** Input parameters (deterministic values and probability distributions) that informed the economic analysis on the cost-effectiveness of an intervention for women aiming at starting and maintaining breastfeeding

Input parameter	Deterministic value	Probability distribution	Source of data – comments
**Characteristics of the modelled population**			
Starting age of women (years)		None	[13]; mean age of women giving birth
- Base-case analysis	30		
- Sensitivity analysis	25, 35		
Mean number of babies per delivery of liveborns	1.016	None	[14]; total number of liveborn babies born to single and multiple maternities were divided by number of maternities that resulted in at least one liveborn
Mean number of babies per woman (used in sensitivity analysis)	1.70	None	[14]; total fertility rate for England
**Intervention specification**			
Effect	1.19	Log-normal: 95% CI 1.10 to 1.30	NICE guideline meta-regression that considered the number of contacts as a variable; effect for 4–8 contacts (in addition to standard care) vs standard care on ‘any breastfeeding between 16 and 26 weeks after birth’ [15]. See Table 1
Cost	£84	Normal: SE = 0.10 of the mean	See Table 2. Distribution based on assumption
**Baseline probability of ‘any breastfeeding’**			
At 4 months	0.42	Beta distribution: α = 418; β = 582	Estimated using the 2019 figure for the prevalence of any breastfeeding at 6–8 weeks [19] and the instant rate of reduction in any breastfeeding between 6 weeks and 4 months, and between 4 months and 6 months, as calculated from 2010 data [20], assuming exponential decrease in breastfeeding rates. See Table 3. Distribution based on assumption.
At 6 months	0.34	Beta distribution: α = 342; β = 658	
**Gastrointestinal infection [GI] in babies – clinical, epidemiological and resource use data**			
Breastfeeding effect (RR) on the incidence of GI	0.46	Log-normal: 95% CI 0.28 to 0.78	[1]; pooled figures for ‘more versus less breastfeeding’, from a mixture of studies with different definitions of the ‘risk factor’ (e.g. exclusive vs non-exclusive; predominant vs partial; partial vs none; any vs none). Effect on incidence of GI from studies in babies and children aged 6 months to 5 years. Effect on incidence of hospitalisation due to GI from studies in babies and children aged < 5 years.
Breastfeeding effect (RR) on the incidence of hospitalisation due to GI	0.28	Log-normal: 95% CI 0.16 to 0.50	
Number of GP consultations for GI in babies aged 0–1 years – current (baseline)	0.047	Beta distribution: α = 47; β = 953	[18]; 4682 GP consultations per 100,000 babies aged < 1 year based on the RCGP database, for the clinical diagnoses of diarrhoea, intestinal infectious diseases, non-infective enteritis, and colitis. Distribution based on assumption.
Hospital admissions for GI in babies aged 0–1 years – current (baseline)	0.015	None	Admissions for babies aged 0–1 years of age for infectious intestinal diseases (ICD10 A00-A09) in England [25], divided by the population aged 0–1 years in England [26].
**Respiratory tract infection [RTI] in babies – clinical, epidemiological and resource use data**			
Breastfeeding effect (RR) on the incidence of lower RTI	0.68	Log-normal: 95% CI 0.60 to 0.77	[1]; pooled figures for ‘more versus less breastfeeding’, from a mixture of studies with different definitions of the ‘risk factor’ (e.g. exclusive vs non-exclusive; predominant vs partial; partial vs none; any vs none). Effects derived from studies in babies and children aged < 2 years
Breastfeeding effect (RR) on the incidence of hospitalisation due to RTI	0.43	Log-normal: 95% CI 0.33 to 0.55	
Number of GP consultations for lower RTI in babies aged 0–1 years – current (baseline)	0.234	Beta distribution: α = 234; β = 766	[18]; 23,433 GP consultations per 100,000 babies aged < 1 year based on the RCGP database, for the clinical diagnosis of lower RTI. Distribution based on assumption
Hospital admissions for RTI in babies aged 0–1 years – current (baseline)	0.115	None	Admissions for babies aged 0–1 years of age for respiratory infectious diseases (ICD10 J00-J22) in England [25], divided by the population aged 0–1 years in England [26].
**Acute otitis media [AOM] in babies – clinical, epidemiological and resource use data**			
Breastfeeding effect (OR) on the incidence of AOM	0.67	Log-normal: 95% CI 0.62 to 0.72	[1]; pooled figures for ‘more versus less breastfeeding’, from a mixture of studies with different definitions of the ‘risk factor’ (e.g. exclusive vs non-exclusive; predominant vs partial; partial vs none; any vs none). Effect derived from studies in babies and children aged ≤2 years
Number of GP consultations for AOM in babies aged 0–1 years – current (baseline)	0.136	Beta distribution: α = 136; β = 864	[18]; 13,556 GP consultations per 100,000 babies aged < 1 year based on the RCGP database, for the clinical diagnosis of AOM. Distribution based on assumption
**Mortality due to infectious diseases and SIDS in babies – clinical and epidemiological data**			
Breastfeeding effect (OR) on mortality due to infectious diseases	0.48	Log-normal: 95% CI 0.30 to 0.60	[1]; pooled figure for ‘any versus never breastfeeding’ Effect derived from studies in babies and children aged 6–23 months
Breastfeeding effect (RR) on mortality due to SIDS	0.38	Log-normal: 95% CI 0.27 to 0.54	[18]; pooled figure for ‘any versus never breastfeeding’. Effect derived from studies in babies and children aged ≥2 months.
Mortality due to infectious diseases in babies aged 0–1 years – current (baseline)	0.00012	None	Number of deaths due to infectious diseases and SIDS in babies aged 0–1 years divided by the number of live births, according to infant mortality data for England and Wales [27].
Mortality due to SIDS in babies aged 0–1 years – current (baseline)	0.00025	None	
Proportion of males among alive babies	0.513	None	Estimated using the number of males and females aged one year in England [26]
**Breast cancer in women – clinical and epidemiological data**			
Breastfeeding effect (OR) on the incidence of breast cancer in parous women	0.86	Log-normal: 95% CI 0.82 to 0.91	[22]; pooled figure for ‘any breastfeeding over 6 months versus never breastfeeding’ adjusted for age, parity, age at first pregnancy, and family history of breast cancer
Effect of parity (OR) on breast cancer - parous women with 2 live births (including previous births) vs non-parous women	0.84	Log-normal: 95% CI 0.80 to 0.89	[31]. The effect was applied onto age-specific incidence of breast cancer in the general population of women (comprising parous and nulliparous women), to get the incidence of breast cancer in parous women
Proportion of nulliparous women		None	[30﻿]
- At 30 years of age	0.48		
- At 35 years of age	0.27		
- At 40 years of age	0.19		
- At 45+ years of age	0.18		
Mean total number of children per parous woman (including previous births)	2	None	[30] (1.90 at 30 years of age, reaching 2.23 at 45 years of age)
Incidence of breast cancer – women in the general population	See Table 1 in Supplementary File 3	None	[29]; see Supplementary File 3 for details
Mortality from breast cancer – women in the general population		None	[32]; see Supplementary File 3 for details
Age-adjusted survival from breast cancer in women over 1–10 years from diagnosis	See Table 2 in Supplementary File 3	None	[33]. After 10 years with breast cancer, women returned to the mortality of the women in the general population, unless they re-developed breast cancer.
**Mortality data**			
Age- and gender-specific mortality in the general population	(multiple data – not shown)	None	[28]
**Utility data**			
Age- and gender-specific utility in the general population	See Table 5	Normal – for SE see Table 5	[34]
Utility in women with breast cancer (years 1–5)	0.67	Beta distribution: α = 67.46; β = 32.54	Estimated from data reported in [35], assuming that the proportion of breast Ca cases that are metastatic at any time is 20%, using information on the proportion of women with metastases at diagnosis (stage IV) [37] and the proportion of women with de novo metastatic disease among prevalent cases of women with metastatic breast cancer [36]. For years 6–10 in breast cancer, utility was the average between the utility of breast cancer and the age-specific utility of women without breast cancer. Distribution based on assumption. See Supplementary File 3 for details.
**Cost data**			
Unit cost of GP visit	£37	Normal: SE = 0.10 of the mean	[16]; cost per consultation lasting 9.22 min, including direct care staff and qualification costs. Distribution based on assumption
Cost per hospital admission for GI	£756	Gamma: SE = 0.10 of the mean	[38]; weighted unit costs for HRG codes PF21A & PF21B, i.e. ‘Paediatric, Infectious or Non-Infectious Gastroenteritis’, with CC Score 1+ and CC Score 0, respectively. Distribution based on assumption
Cost per hospital admission for RTI	£1094	Gamma: SE = 0.10 of the mean	[38]; weighted unit costs for HRG codes PD11, Paediatric, Acute Upper Respiratory Tract Infection or Common Cold, with CC Score 0 to 4+, PD14, Paediatric Lower Respiratory Tract Disorders without Acute Bronchiolitis, with CC Score 0 to 11+, PD15, Paediatric Acute Bronchiolitis with CC Score 0 to 5+, PD65, Paediatric Upper Respiratory Tract Disorders with CC Score 0 to 5+, and PD12, Paediatric, Asthma or Wheezing, with CC Score 0 to 4+. Distribution based on assumption
Unit cost of death due to an infectious disease or SIDS in babies	£204	Gamma: SE = 0.10 of the mean	[38]; unit cost for HRG code VB99Z ‘Emergency medicine, patient dead on arrival’. Distribution based on assumption
Post-mortem cost added in sensitivity analysis	£8000		[39]; comprising the cost of a paediatric coronial case of about £2000 and the cost of a forensic examination of about £6000
Healthcare cost in women with breast cancer and those without breast cancer	See Table 3 in Supplementary File 3	Gamma: SE = 0.10 of the mean	[40]; data on 359,771 women with breast cancer. Distribution based on assumption; see Supplementary File 3 for details.
**Annual discount rate**			
- base-case analysis	0.035	None	[41]; applied to both costs and outcomes.
- sensitivity analysis	0.015	None	

*AOM* acute otitis media, *CI* confidence intervals, *GI* gastrointestinal infection, *GP* general practitioner, *HRG* hospital related group, *OR* odds ratio, *RCGP* Royal College of General Practitioners, *RR* risk ratio, *RTI* respiratory tract infection, *SE* standard error, *SIDS* sudden infant death syndrome

In addition, one-way sensitivity analysis explored the following scenarios:

- Attaching a post-mortem cost of £8000 to each baby’s death caused by an infectious disease or SIDS
- Assuming that women received their intervention when having their first baby and that the effect of the intervention was retained in future births, thus the benefits of breastfeeding applied to all future babies born by each woman receiving the intervention (in total 1.70 babies per woman [14]). For this scenario, we also assumed that the total duration of breastfeeding across pregnancies was 12 months; however, despite of the dose-response relation between breastfeeding duration and breast cancer risk in parous women, the effect of breastfeeding on the incidence of breast cancer was the same between a 6- and a 12-month duration [22]
- Use of an annual discount rate of 1.5%
- Changing the starting age of the cohort of mothers by ±5 years (25 and 35 years of age).

### Validation of the economic model

The economic model was developed in collaboration with members of the guideline committee, using a previous economic model [18] as a basis. All inputs and model formulae were systematically checked. The model was tested for logical consistency by setting input parameters to null and extreme values and examining whether results changed in the expected direction. Results were discussed with the committee to confirm their plausibility. Moreover, where modelling structure components were identical to those of Renfrew et al. [18], input data from that study were used to confirm that its results could be replicated using our model.

## Results

Table 7 shows the results of the base-case economic analysis. The table provides the total intervention cost as well as total costs and outcomes associated with every clinical condition considered in the economic analysis, for 1000 women and their babies. The intervention had better outcomes and resulted in cost-savings across all conditions examined, when added on standard care compared with standard care alone. Overall, it was more costly than its comparator as the cost-savings resulting from provision of the intervention were not adequate to offset the intervention costs. The ICER of the intervention added on standard care versus standard care alone was £51,946/QALY, which is well above the NICE upper cost-effectiveness threshold of £30,000/QALY [43], suggesting that the intervention is not cost-effective. The table shows the results of the deterministic analysis, as these are directly comparable to the results of the two-way sensitivity analysis. Results of deterministic and probabilistic sensitivity analysis were very similar; the ICER of the probabilistic analysis was £51,639/QALY.

**Table 7:** Results of base-case economic analysis: cost-effectiveness of interventions aiming at promoting breastfeeding (results for 1000 women and their babies)

Parameter		Intervention + SC	SC alone	Difference
Intervention cost		£84,000	£0	£84,000
GI in babies	Infections	44.91	47.56	−2.65
	Hospitalisations	14.27	15.55	−1.28
	Costs	£12,469	£13,535	-£1066
(lower) RTI in babies	Infections	231.01	238.04	−7.02
	Hospitalisations	110.26	117.27	−7.01
	Costs	£129,272	£137,204	-£7932
AOM in babies	Infections	133.49	137.70	−4.21
	Costs	£4993	£5150	-£157
Mortality in babies due to infections and SIDS	Deaths due to infections	0.11	0.12	−0.01
	Deaths due to SIDS	0.24	0.25	−0.02
	Costs of deaths prevented	-£1		-£1
	QALYs gained	0.16		0.16
Breast cancer in women	New cases	138.35	139.65	−1.29
	QALYs	20,945.72	20,944.63	1.09
	Costs	£7,033,056	£7,043,111	-£10,056
**Total difference in QALYs**				1.25
**Total difference in costs**				£64,787
**ICER**		**£51,946/QALY**		

*AOM* acute otitis media, *GI* gastrointestinal infection, *ICER* incremental cost-effectiveness ratio, *RTI* respiratory tract infection, *SC* standard care, *SIDS* sudden infant death syndrome

Results of two-way sensitivity analysis are shown in Table 8, for different combinations of intervention effect and intervention cost. Result cells with bold content show combinations for which the intervention is cost-effective, with an ICER below the NICE lower cost-effectiveness threshold of £20,000/QALY, or with the intervention being dominant when added onto standard care, i.e. being both more effective and less costly compared with standard care alone. Result cells with figures in italics show combinations where the ICER is between £20,000–£30,000/QALY. All other result cells show combinations for which the intervention is not cost-effective, with an ICER above the NICE upper cost-effectiveness threshold of £30,000/QALY. The underlined figures show the intervention cost and effect values used in base-case analysis and the base-case ICER.

**Table 8:** Results of two-way sensitivity analysis: cost-effectiveness of interventions aiming at promoting breastfeeding

		Intervention cost																
		£20	£25	£30	£35	£40	£45	£50	£55	£60	£65	£70	£75	£80	£84	£90	£95	£100
**Intervention effect**	**1.05**	£45,852	£61,166	£76,480	£91,795	£107,109	£122,423	£137,737	£153,052	£168,366	£183,680	£198,994	£214,309	£229,623	£241,874	£260,251	£275,566	£290,880
	**1.10**	**£15,224**	*£22,881*	£30,538	£38,195	£45,852	£53,509	£61,166	£68,823	£76,480	£84,138	£91,795	£99,452	£107,109	£113,235	£122,423	£130,080	£137,737
	**1.15**	**£5014**	**£10,119**	**£15,224**	*£20,328*	*£25,433*	£30,538	£35,642	£40,747	£45,852	£50,957	£56,061	£61,166	£66,271	£70,355	£76,480	£81,585	£86,690
	**1.19**	**£631**	**£4640**	**£8649**	**£12,658**	**£16,667**	*£20,676*	*£24,685*	*£28,694*	£32,703	£36,712	£40,721	£44,729	£48,738	£51,946	£56,756	£60,765	£64,774
	**1.25**	**dominant**	**dominant**	**£2972**	**£6035**	**£9098**	**£12,161**	**£15,224**	**£18,286**	*£21,349*	*£24,412*	*£27,475*	£30,538	£33,601	£36,051	£39,726	£42,789	£45,852
	**1.30**	**dominant**	**dominant**	**dominant**	**£2462**	**£5014**	**£7566**	**£10,119**	**£12,671**	**£15,224**	**£17,776**	*£20,328*	*£22,881*	*£25,433*	*£27,475*	£30,538	£33,090	£35,642
	**1.35**	**dominant**	**dominant**	**dominant**	**dominant**	**£2097**	**£4285**	**£6473**	**£8660**	**£10,848**	**£13,036**	**£15,224**	**£17,411**	**£19,599**	*£21,349*	*£23,974*	*£26,162*	*£28,350*
	**1.40**	**dominant**	**dominant**	**dominant**	**dominant**	**dominant**	**£1824**	**£3738**	**£5652**	**£7566**	**£9481**	**£11,395**	**£13,309**	**£15,224**	**£16,755**	**£19,052**	*£20,966*	*£22,881*
	**1.45**	**dominant**	**dominant**	**dominant**	**dominant**	**dominant**	**dominant**	**£1611**	**£3312**	**£5014**	**£6716**	**£8417**	**£10,119**	**£11,820**	**£13,182**	**£15,224**	**£16,925**	**£18,627**
	**1.50**	**dominant**	**dominant**	**dominant**	**dominant**	**dominant**	**dominant**	**dominant**	**£1441**	**£2972**	**£4504**	**£6035**	**£7566**	**£9098**	**£10,323**	**£12,161**	**£13,692**	**£15,224**
	**1.55**	**dominant**	**dominant**	**dominant**	**dominant**	**dominant**	**dominant**	**dominant**	**dominant**	**£1301**	**£2694**	**£4086**	**£5478**	**£6870**	**£7984**	**£9655**	**£11,047**	**£12,439**
	**1.60**	**dominant**	**dominant**	**dominant**	**dominant**	**dominant**	**dominant**	**dominant**	**dominant**	**dominant**	**£1185**	**£2462**	**£3738**	**£5014**	**£6035**	**£7566**	**£8843**	**£10,119**
	**1.65**	**dominant**	**dominant**	**dominant**	**dominant**	**dominant**	**dominant**	**dominant**	**dominant**	**dominant**	**dominant**	**£1087**	**£2265**	**£3443**	**£4386**	**£5799**	**£6977**	**£8155**
	**1.70**	**dominant**	**dominant**	**dominant**	**dominant**	**dominant**	**dominant**	**dominant**	**dominant**	**dominant**	**dominant**	**dominant**	**£1003**	**£2097**	**£2972**	**£4285**	**£5379**	**£6473**
	**1.75**	**dominant**	**dominant**	**dominant**	**dominant**	**dominant**	**dominant**	**dominant**	**dominant**	**dominant**	**dominant**	**dominant**	**dominant**	**£930**	**£1747**	**£2972**	**£3993**	**£5014**
	**1.80**	**dominant**	**dominant**	**dominant**	**dominant**	**dominant**	**dominant**	**dominant**	**dominant**	**dominant**	**dominant**	**dominant**	**dominant**	**dominant**	**£675**	**£1824**	**£2781**	**£3738**
	**1.85**	**dominant**	**dominant**	**dominant**	**dominant**	**dominant**	**dominant**	**dominant**	**dominant**	**dominant**	**dominant**	**dominant**	**dominant**	**dominant**	**dominant**	**£810**	**£1711**	**£2612**
	**1.90**	**dominant**	**dominant**	**dominant**	**dominant**	**dominant**	**dominant**	**dominant**	**dominant**	**dominant**	**dominant**	**dominant**	**dominant**	**dominant**	**dominant**	**dominant**	**£760**	**£1611**
	**1.95**	**dominant**	**dominant**	**dominant**	**dominant**	**dominant**	**dominant**	**dominant**	**dominant**	**dominant**	**dominant**	**dominant**	**dominant**	**dominant**	**dominant**	**dominant**	**dominant**	**£715**
	**2.00**	**dominant**	**dominant**	**dominant**	**dominant**	**dominant**	**dominant**	**dominant**	**dominant**	**dominant**	**dominant**	**dominant**	**dominant**	**dominant**	**dominant**	**dominant**	**dominant**	**dominant**

Result cells with bold content: results where ICER < £20,000/QALY; or where intervention + standard care is dominant = less costly and more effective than standard care alone

Result cells with content in italics: results where ICER is between £20,000–£30,000/QALY (lower - upper NICE cost-effectiveness threshold)

All other result cells: results where ICER > £30,000/QALY

Underlined figures: intervention cost and effect values used in the base-case analysis; result of the base-case analysis

As expected, the cost-effectiveness of the intervention improves as its effectiveness increases and its intervention cost decreases. At the base-case relative effect (RR) of 1.19 (for any breastfeeding at 16–26 weeks after birth), the intervention becomes cost-effective (<£20,000/QALY) if its cost per woman receiving the intervention is approximately £40–£45. On the other hand, at the base-case cost of £84, the intervention becomes cost-effective if its effectiveness (in terms of breastfeeding rates), when added on standard care, is at least 35–40% higher than the effectiveness of standard care alone (i.e. if the RR reaches 1.35–1.40).

Table 9 shows results of other scenarios tested in sensitivity analysis. Inclusion of the post-mortem examination cost for babies dying due to an infectious disease or SIDS had practically no impact on the ICER, which was expected, given the very low mortality due to an infectious disease or SIDS in babies aged 0–1 years. Similarly, assuming that all women received the intervention when they had their first baby and that the effect of the intervention on breastfeeding rates would be retained in all subsequent births had no impact on our conclusion. On the other hand, results were considerably affected by the use of an annual discount rate of 1.5% for both costs and outcomes, with the ICER falling at a value between the lower and upper NICE cost-effectiveness threshold of £20,000–£30,000/QALY. This finding is explained by the fact that the greatest part of clinical benefits of breastfeeding (prevention of breast cancer) is enjoyed by women several years after breastfeeding takes place (as the incidence of breast cancer in women considerably increases after the age of 40 years), so reducing the discount factor places a greater value on the benefits and cost-savings accrued in the long-term. Because of discounting, reducing the starting age of women cohort at 25 years increased the ICER (as QALY gains resulting from prevention of breast cancer occurred after a longer period of time compared with the base-case analysis and were thus placed a lower value due to discounting), whereas increasing the starting age of women at 35 years reduced the ICER (because prevention of breast cancer occurred sooner compared with the base-case analysis and was thus placed a higher value). Since women aged ≤35 years represent 78% of women giving birth in England and Wales [13], the intervention is unlikely to be cost-effective under NICE criteria for the whole study population, even if it is found to be cost-effective in women giving birth aged beyond 35 years.

**Table 9:** Results of alternative scenarios tested in sensitivity analysis

Scenario tested in sensitivity analysis	ICER
Inclusion of post-mortem examination cost for baby deaths	£51,904/QALY
Assuming effect of intervention is retained in future births, so that breastfeeding benefits apply to all babies born to a woman	£43,223/QALY
Use of an annual discount rate of 1.5%	£22,667/QALY
Starting age of women 25 years	£60,145/QALY
Starting age of women 35 years	£46,068/QALY

## Discussion

### Overview and interpretation of findings

The results of our analysis suggest that an intervention aimed at promoting breastfeeding, which comprises provision of information and support to women by healthcare workers and/or breastfeeding peer supporters, initiated in the antenatal period or up to 8 weeks after birth, is unlikely to be cost-effective, as its ICER when added onto standard care versus standard care alone was £51,946/QALY, which is well above the NICE upper cost-effectiveness threshold of £30,000/QALY. Results of sensitivity analysis suggested that the intervention might become cost-effective if its cost was reduced by about 50% (from £84 to around £40–£45 per woman receiving the intervention) or if its relative effect (RR) on any breastfeeding 16–26 weeks after birth was improved by about 100% (from 1.19, i.e. 19% increase, to 1.35–1.40, i.e. 35–40% increase in breastfeeding rates). Translating the intervention effect into numbers of women breastfeeding, in a cohort of 100 women, 42 would breastfeed under standard care (estimate based on national statistics), 50 would breastfeed if the modelled intervention with a RR of 1.19 was offered to the cohort, and 57 women in the cohort would need to breastfeed (estimated using a RR of 1.36) for the intervention to become cost-effective.

A less resource intensive intervention comprising two individual 30-min sessions provided by a health professional in NHS England AfC Band 5 has a cost of £59, which is higher than the cost of £40–45 that would be required for the intervention to be cost-effective; moreover, according to our meta-regression, an intervention comprising two contacts would only be expected to have a small and non-significant effect (RR 1.07, 95% CI 0.98 to 1.17, for 2–3 contacts versus standard care). An intervention cost of £40, required for the intervention to become cost-effective, could be achieved by 4 individual 30-min sessions provided by a peer supporter, assuming a unit cost of £20 per hour. However, it is possible that the unit cost of a peer supporter is higher, if childcare costs are taken into account, meaning that an intervention cost as low as £40 may not be achievable even by provision of the intervention by a peer supporter offering 4 individual 30-min sessions.

Furthermore, the RR of 1.35–1.40 that would be required for the intervention to become cost-effective is above the upper 95% CI of the relative effect used in the base-case analysis (mean RR 1.19, 95% CI 1.10 to 1.30). Therefore, it appears that an intervention of this type needs to be both more effective and less costly than its specification in our economic analysis, for it to be cost-effective within the NICE decision-making context.

### Strengths and limitations

The effectiveness of the intervention in improving breastfeeding rates was determined by a meta-regression of RCTs [15] conducted to inform the NICE guideline on postnatal care [12]. The quality of the included data was overall low. Typically, the studies included in the review were characterised by serious risk of bias associated with the randomisation process, selective reporting and missing outcome data, as assessed using the Cochrane Risk of Bias tool [44]. Most studies were unblinded, which was unsurprising given the nature of the interventions. Interventions included in the review were characterised by particularly high heterogeneity regarding the mode of delivery (e.g. face-to-face or by telephone, individually or in groups, blended interventions were also common), the number of contacts and the duration of the intervention, the place of delivery (at home, in hospital, in a community setting or a combination of locations), the person delivering the intervention (peer supporter, lactation consultant, midwife, health visitor or a combination), and the involvement of fathers. Moreover, there was heterogeneity regarding the study participants’ intention to breastfeed at recruitment, and the definition of ‘any breastfeeding’ as an outcome. Standard care was also heterogeneous and, in general, poorly described.

The structure of the economic model and the estimates of the association of breastfeeding with clinical outcomes were based on high quality studies [1, 18] identified from a systematic review of the literature conducted specifically to inform our analysis. However, the primary data on the association of breastfeeding with clinical outcomes that were synthesised in meta-analysis were derived from study designs that were prone to bias; several studies demonstrating clinical benefits associated with breastfeeding had adjusted for some known confounders. Nevertheless, it is possible that there are other unknown confounders impacting on the relation between breastfeeding and clinical benefits, for which the studies were unable to adjust. Moreover, other studies made no adjustments for confounding. Thus, it is possible that the magnitude of the clinical benefits of breastfeeding have been overestimated in this literature and, consequently, in our analysis.

One further point to note is that evidence on the association between breastfeeding and mortality from infectious diseases was derived exclusively from low and medium income countries due to lack of high income country data, so findings may not be directly relevant to the population in the UK. However, the impact of this input parameter on the results was negligible.

Breastfeeding has been found to be associated with several clinical conditions that were not possible to consider in our economic model, either due to lack of suitable and/or good quality epidemiological and cost data that would allow robust modelling to be conducted, or due to the complexity or uncertainty of modelling owing to the multifactorial nature of some diseases. For example, breastfeeding has been associated with a reduced risk of diabetes in both mothers and babies and a reduced risk of obesity in babies over their lifetime. It has also been associated with improved cognitive outcomes in babies and reduced incidence of ovarian cancer in mothers [1]. Furthermore, there is some evidence that breastfeeding has a protective effect on the development of triple negative breast cancer [45, 46], which is considered to be more aggressive and have a poorer prognosis compared with other types of breast cancer. Current evidence suggests a protective effect of breastfeeding on the incidence of and hospitalisations due to GI in babies over the first 5 years of their life, and on the incidence of lower RTI and AOM, as well as hospitalisations due to RTI over the first 2 years of their life [1]. We conservatively modelled respective cost-savings and benefits to babies only over the first year of their lives, due to the availability of relevant epidemiological data. Prevention of infections in babies, which is associated with breastfeeding, results in lower antibiotic use and thus lower rates of antimicrobial resistance in the community. Mothers who wish to breastfeed but experience societal barriers or lack of skilled support may experience psychological distress if they are not able to breastfeed. A successful breastfeeding intervention that enables them to breastfeed is likely to improve their mental wellbeing and promote emotional attachment with their baby, improving also the baby’s psychological development. Such benefits were not captured in our analysis.

Clinical benefits such as the reduction in the incidence of GI, RTI and AOM in babies were not translated into QALYs and thus were not considered in the estimation of the ICER. On the other hand, QALY gains associated with these benefits are expected to be very small due to the usually short duration of these outcomes (only a few days or weeks). Equally, the ICER has not captured the intangible benefits to parents associated with improved outcomes in babies, in particular the psychological burden avoided by a reduction in mortality due to infectious diseases or SIDS.

Benefits were only estimated for healthy babies at term. Breastfeeding has been shown to be associated with clinical benefits to premature, low-birth-weight, or seriously ill babies [47–50]. Such benefits were not captured by our analysis as they were beyond its scope.

The clinical data on the protective effect of breastfeeding on the incidence of breast cancer expressed the difference in the incidence of breast cancer between parous women that breastfed over 6 months after birth and those who never breastfed [22]; similarly, the clinical data on the protective effect of breastfeeding on mortality due to infectious diseases or SIDS in babies expressed the difference in mortality between babies that were breastfed (at 4 months after birth) and those that were never breastfed. A shorter duration of breastfeeding (e.g. 2–3 months) is possible to still have a protective effect on breast cancer in women [22] and death from infectious diseases or SIDS in babies. However, our model considered the effectiveness of the breastfeeding intervention regarding the breastfeeding status of women and babies at a single time point (4 months after birth for babies, 6 months for women). Some of the women who were not breastfeeding at 6 months may have breastfed until an earlier time point (i.e. they are not necessarily women who never breastfed their babies from birth to 6 months); similarly, some of the babies who were not breastfed at 4 months may have been breastfed until an earlier time point (i.e. they are not necessarily babies who were never breastfed from birth and up to 4 months); these women and babies have received some protective effect from breastfeeding in reducing the incidence of breast cancer and mortality due to infectious diseases or SIDS, respectively. In this aspect, our analysis has likely overestimated the benefits and cost-savings of breastfeeding (and, consequently, of the breastfeeding intervention) to mothers and babies.

As infant mortality from infectious diseases or SIDS is rare, the overestimation of the protective effect of breastfeeding is expected to be negligible and thus highly unlikely to have impacted on the results and conclusions of the analysis. In contrast, the overestimation of the protective effect of breastfeeding on the reduction in the incidence of breast cancer has potentially a significant impact on the model results, given that the QALYs and cost-savings from the reduction in the incidence of breast cancer contributed considerably to the estimation of the ICER (QALYs gained due to a reduction in the incidence of breast cancer accounted for 95% of total QALYs gained following provision of the breastfeeding intervention; cost-savings due to a reduction in the incidence of breast cancer accounted for 51% of the total cost-savings following provision of the breastfeeding intervention).

According to an older high-quality meta-analysis of 47 epidemiological studies in 30 countries [17], the impact of any versus no breastfeeding for up to 6 months on breast cancer is very small and non-significant (OR 0.98, 95% CI 0.95 to 1.01), while the impact of any versus no breastfeeding for a duration of 7–18 months is statistically significant (OR 0.94, 95% CI 0.91 to 0.97), but smaller than the estimate from the meta-analysis that informed our economic analysis [22]. The two meta-analyses included different studies and reported rather contradictory results on whether breastfeeding for a duration of up to 6 months can reduce the incidence of breast cancer. Results of the older meta-analysis also suggest that our model may have overestimated the clinical benefits and associated cost-savings of the breastfeeding intervention in relation to the reduction in the incidence of breast cancer. It is noted, though, that Victora et al. [1] reported the results of a meta-analysis, according to which highest versus lowest duration of breastfeeding showed a larger and statistically significant protective effect on the incidence of breast cancer (OR 0.81, 95% CI 0.77 to 0.86). However, most studies in this meta-analysis had not adjusted appropriately for parity and therefore tended to exaggerate the effect size.

The guideline committee expressed the view that, overall, the magnitude of overestimation of benefits and cost-savings in some aspects of the economic analysis balanced out the magnitude of the underestimation of benefits and cost-savings in other areas, where modelling was limited or not conducted. After considering the strengths and the weaknesses of our analysis, the committee concluded that, currently, providing an education and support intervention, in addition to standard care, that aims to promote breastfeeding to women that are pregnant or have given birth to healthy babies at term, does not appear to be cost-effective in the UK under NICE criteria of cost-effectiveness.

### Generalisability of the results and implications of the study

Our analysis was conducted from the perspective of the NHS/PSS in England. Results may be generalisable to other settings with similar funding and structure of healthcare and personal social services, including postnatal care pathways, as well as comparable epidemiological picture regarding the clinical conditions associated with breastfeeding. Interventions for mothers, aimed at promoting breastfeeding, may be considerably more cost-effective in settings where the baseline risk of infections to babies and/or breast cancer to mothers, and/or associated management costs, are higher compared with the UK, for example, in low and middle-income country settings. We also note that our results were sensitive to the discount rate used, as use of a lower discount rate led to the intervention’s cost-effectiveness being considerably improved. The choice of the discount rate for costs and outcomes in economic evaluations of health care programmes and policies has been contentious and the subject of ongoing discussion [51–54] and may have a stronger impact in economic evaluations of strategies with long-term consequences, such as prevention programmes, which are disfavoured by use of higher discount rates [52, 53]. Conclusions on cost-effectiveness ultimately rely on the perspective of the analysis and the cost-effectiveness threshold adopted; the latter depends on the policy makers’ willingness-to-pay for clinical and other wider benefits, which may vary across countries and health systems.

As other literature suggests, worldwide, breastfeeding itself is cost-effective as it leads to important clinical benefits to mothers and babies and cost-savings to the health service, parents and the whole society, at no intervention cost [18, 21, 55–60]. Our economic analysis only demonstrated that the breastfeeding intervention, as specified in our model, was not cost-effective when added to standard care because the clinical benefits and cost-savings resulting from an increase in breastfeeding rates, although important, were not adequate to outweigh initial intervention costs. This is because the baseline incidence of the clinical conditions assessed in the model is already rather low in the general population of women giving birth to healthy babies, and their babies, in the UK compared with other settings, e.g. the rate of baby infections is higher in low-income countries and therefore clinical benefits to babies and associated cost-savings resulting from breastfeeding (i.e. a reduction in baby infection rates and subsequent hospitalisations) are expected to be larger. Moreover, the effectiveness of the intervention in improving breastfeeding rates at 16–26 weeks was relatively small (mean RR of the intervention added onto standard care versus standard care alone 1.19), with a rather small impact at a population level. To put this figure into context, out of 100 women receiving the intervention, 42 would breastfeed at 4 months under standard care whether they received the intervention or not (according to national statistics), and only 8 would breastfeed because of the intervention (19% of 42 according to the intervention’s effectiveness). Therefore, the benefit of the intervention offered to 100 women would be 8 additional women breastfeeding. At an intervention cost of £84 per woman receiving the intervention, it would cost £8400 to support 8 extra women to breastfeed, or £1050 per extra woman breastfeeding.

Future research should focus on the effective components of breastfeeding interventions, as identified by this meta-regression, aiming at the development of breastfeeding interventions with higher effectiveness that are less resource intensive and offer more return on investment than currently available interventions. Women’s needs and views on facilitators and barriers to breastfeeding need also to be taken into account when designing such interventions.

Perhaps the way forward to improve breastfeeding rates in a cost-effective way is to implement public health and other societal interventions, which do not target women specifically but apply changes within society as a whole to promote and normalise breastfeeding, after identifying and targeting factors that dissuade mothers from breastfeeding in current UK society, for example, the pressures of advertising by infant formula companies, negative public attitudes to breastfeeding in public, and the lack of appropriate facilities for breastfeeding mothers. Such interventions could include, for example, full adoption of the WHO International Code of marketing of breast milk substitutes [61], further improvements to maternity leave arrangements and pay, and other workplace policies that enable working women to breastfeed their babies. Such interventions were beyond the scope of the NICE updated guideline on postnatal care, and therefore we have not explored their effects and cost-effectiveness. It is also possible that, if some of the barriers to breastfeeding are removed by implementation of societal interventions, this will lead to an improvement of the effectiveness of education and support interventions targeted to pregnant women and those who have given birth.

Based on the results of this meta-regression and economic analysis, the guideline committee was unable to recommend specific education and support breastfeeding interventions of the type assessed in the economic analysis; instead, they made recommendations that aim to enforce national breastfeeding initiatives and optimise current NHS postnatal care in England, to improve the quality and reduce variation in the current provision of breastfeeding advice and support across settings.

## Conclusion

Worldwide, breastfeeding results in health benefits to women and their babies and cost-savings to the health service. Available evidence on antenatal and postnatal breastfeeding interventions for women, which comprise education, advice and support from a mixture of health professionals and peer volunteers, suggests these may not be cost-effective when added to standard care under NICE criteria in England. More effort needs to be placed on how breastfeeding education, advice and support can be optimised in order to improve the quality and reduce variation in services offered across care settings. Public health and other societal interventions that facilitate breastfeeding and remove barriers to it may be key in improving breastfeeding rates in the UK.

## Supplementary Information

**Additional file 1.** Systematic review of studies that modelled long-term clinical benefits to mothers and babies (and/or related cost-savings to health and personal social services) associated with breastfeeding: included studies and studies excluded after full text was obtained.

**Additional file 2.** Evidence on clinical conditions associated with breastfeeding that was considered in the development of the economic analysis on the cost-effectiveness of breastfeeding interventions.

**Additional file 3.** Age-specific incidence of breast cancer, mortality, cost and utility data that informed the economic model component on women’s breast cancer.

## Data Availability

All data generated or analysed during this study are included in this published article and its supplementary information files.

## Abbreviations

AfC: Agenda for change
AOM: Acute otitis media
CI: Confidence intervals
GI: Gastrointestinal infection
GP: General practitioner
ICER: Incremental cost-effectiveness ratio
NHS: National health service
NICE: National Institute for Health and Care Excellence
OR: Odds ratio
PSS: Personal social services
QALY: Quality-adjusted life year
RCGP: Royal College of General Practitioners
RCT: Randomised controlled trial
RR: Risk ratio
RTI: Respiratory tract infection
SIDS: Sudden infant death syndrome
WHO: World Health Organization
