# Supplementary material for: The cost-effectiveness of antenatal and postnatal education and support interventions for women aimed at promoting breastfeeding in the UK
Source: BMC Public Health. 2022 Jan 22;22:153. doi: 10.1186/s12889-021-12446-5 (PMC8783468; doi:10.1186/s12889-021-12446-5)
Supplement: Supplementary file 1 — Additional file 1. Systematic review of studies that modelled long-term clinical benefits to mothers and babies (and/or related cost-savings to health and personal social services) associated with breastfeeding: included studies and studies excluded after full text was obtained. [file 12889_2021_12446_MOESM1_ESM.docx]

**Supplementary File 1.**

**Systematic review of studies that modelled long-term clinical benefits to mothers and babies (and/or related cost-savings to health and personal social services) associated with breastfeeding: included studies and studies excluded after full text was obtained.**

### List of included modelling studies and overview of study characteristics of interest

| **Included study full reference** | **Country and outcomes considered** |
| --- | --- |
| Bartick M, Reinhold A. The burden of suboptimal breastfeeding in the United States: a pediatric cost analysis. Pediatrics 2010; 125(5): e1048-56. | US study  Outcomes for the baby: NEC, otitis media, gastroenteritis, hospitalisation for lower respiratory tract infection, atopic dermatitis, SIDS, childhood leukaemia, childhood asthma, type 1 diabetes mellitus, obesity.  Study was considered by Renfrew et al. 2012 |
| Bartick M. Breastfeeding and the U.S. economy. Breastfeed Med 2011; 6: 313-8. | US study  Further analysis to Bartick et al. 2010; additional data on formula feeding costs, cost of extra food for lactating women, paid leave. |
| Bartick MC, Stuebe AM, Schwarz EB, Luongo C, Reinhold AG, Foster EM. Cost analysis of maternal disease associated with suboptimal breastfeeding. Obstet Gynecol 2013; 122(1): 111-9. | US study  Outcomes for the mother: breast cancer, ovarian cancer, hypertension, type 2 diabetes, myocardial infarction.  Data sources were either considered in Renfrew et al. 2012 or included in systematic reviews reported by Victora et al. 2016 |
| Bartick MC, Schwarz EB, Green BD, Jegier BJ, Reinhold AG, Colaizy TT, Bogen DL, Schaefer AJ, Stuebe AM. Suboptimal breastfeeding in the United States: Maternal and pediatric health outcomes and costs. Maternal and Child Nutrition 2017a; 13(1). | US study  Outcomes for the baby: acute lymphoblastic leukaemia, acute otitis media, Crohn's disease, ulcerative colitis, gastrointestinal infection, lower respiratory tract infection requiring hospitalisation, obesity, NEC, SIDS.  Outcomes for the mother: breast cancer, pre‐menopausal ovarian cancer, hypertension, type 2 diabetes, myocardial infarction.  Data sources were either considered in Renfrew et al. 2012 or included in systematic reviews reported by Victora et al. 2016 |
| Bartick MC, Jegier BJ, Green BD, Schwarz EB, Reinhold AG, Stuebe AM. Disparities in Breastfeeding: Impact on Maternal and Child Health Outcomes and Costs. J Pediatr 2017b; 181: 49-55.e6. | US study  Sub-group analysis of Batrick et al. 2017a. |
| Bartick M. Mothers' costs of suboptimal breastfeeding: implications of the maternal disease cost analysis. Breastfeed Med. 2013; 8(5):448-9. | US study  Secondary analysis of Bartick et al. 2013; cost analysis of maternal disease associated with suboptimal breastfeeding. |
| Büchner FL, Hoekstra J, van Rossum CTM. Health gain and economic evaluation of breastfeeding policies: Model simulation. Bilthoven, Netherlands: RIVM, 2007. | Dutch study.  Outcomes for the baby: gastrointestinal infection, otitis media, respiratory infection, asthma, eczema, Crohn’s disease, obesity, leukaemia.  Outcomes for the mother: breast cancer, ovarian cancer, rheumatoid arthritis for the mother.  Study was considered by Renfrew et al. 2012 |
| Chola L, Fadnes LT, Engebretsen IM, Nkonki L, Nankabirwa V, Sommerfelt H, Tumwine JK, Tylleskar T, Robberstad B; PROMISE-EBF Study Group. Cost-Effectiveness of Peer Counselling for the Promotion of Exclusive Breastfeeding in Uganda. PLoS One 2015; 10(11):e0142718. | Ugandan study  Outcomes for the baby: diarrhoea.  Data on association between breastfeeding and diarrhoea based on SR of studies in developing countries. |
| Colchero MA, Contreras-Loya D, Lopez-Gatell H, González de Cosío T. The costs of inadequate breastfeeding of infants in Mexico. Am J Clin Nutr 2015; 101(3):579-86. | Mexican study  Outcomes for the baby: respiratory infection, otitis media, gastroenteritis, NEC, SIDS.  Data sources on association between breastfeeding and diarrhoea were considered in Renfrew et al. 2012 |
| Ma P, Brewer-Asling M, Magnus JH. A case study on the economic impact of optimal breastfeeding. Matern Child Health J. 2013; 17(1):9-13. | US study  Outcomes for the baby: respiratory tract infection, gastroenteritis, NEC, SIDS.  Data sources on association between breastfeeding and the 4 infant diseases were considered in Renfrew et al. 2012 |
| McIsaac KE, Moineddin R, Matheson FI. Breastfeeding as a means to prevent infant morbidity and mortality in Aboriginal Canadians: A population prevented fraction analysis. Can J Public Health. 2015; 106(4):e217-22. | Canadian study  Outcomes for the baby: SIDS, gastrointestinal infection, respiratory tract infection, otitis media.  Data sources on association between breastfeeding and the outcomes for the baby were considered in Renfrew et al. 2012 |
| Pokhrel S, Quigley MA, Fox-Rushby J, McCormick F, Williams A, Trueman P, Dodds R, Renfrew MJ. Potential economic impacts from improving breastfeeding rates in the UK. Arch Dis Child 2015; 100(4): 334-40. | UK study  Secondary publication to Renfrew et al. 2012 |
| Renfrew M, Pokhrel S, Quigley M, et al. Preventing disease and saving resources: the potential contribution of increasing breastfeeding rates in the UK. London: Unicef UK, 2012. | UK study  Outcomes for the baby: gastrointestinal infection, respiratory tract infection, otitis media, NEC  Outcomes for the mother: breast cancer  In addition, narrative description of economic benefits for the following outcomes for the baby: SIDS, cognitive outcomes, obesity  Epidemiological and resource use data relevant to guideline analysis |
| Rollins NC, Bhandari N, Hajeebhoy N, Horton S, Lutter CK, Martines JC, Piwoz EG, Richter LM, Victora CG, Lancet Breastfeeding Series Group. Why invest, and what it will take to improve breastfeeding practices? Lancet 2016; 387(10017):491-504. | Global analysis  Outcomes for the baby: diarrhoea, pneumonia, bronchiolitis, NEC, otitis media, asthma, leukaemia, type 1 diabetes, obesity, cognitive outcomes  Data on association between breastfeeding and outcomes for the baby were obtained from Victora et al. 2016; epidemiological and resource use data for the UK were obtained from Renfrew et al. 2012. |
| Stuebe AM, Jegier BJ, Schwarz EB, Green BD, Reinhold AG, Colaizy TT, Bogen DL, Schaefer AJ, Jegier JT, Green NS, Bartick MC. An Online Calculator to Estimate the Impact of Changes in Breastfeeding Rates on Population Health and Costs. Breastfeed Med 2017; 12(10):645-658 | US study  Estimation of population benefits and costs was made using the model and data reported by Bartick et al. 2017 |
| Straub N, Grunert P, Northstone K, Emmett P. Economic impact of breast-feeding-associated improvements of childhood cognitive development, based on data from the ALSPAC. Br J Nutr 2016; 22:1-6. | UK study  Outcomes for the baby: attainment at school KS4, linked to individual gross income |
| Unar-Munguía M, Meza R, Colchero MA, Torres-Mejía G, de Cosío TG. Economic and disease burden of breast cancer associated with suboptimal breastfeeding practices in Mexico. Cancer Causes Control 2017; 28(12): 1381-1391. | Mexican study  Outcomes for the mother: breast cancer  Data source for the association between breastfeeding and breast cancer: Unar-Munguía M, Torres-Mejía G, Colchero MA, González de Cosío T. Breastfeeding Mode and Risk of Breast Cancer: A Dose-Response Meta-Analysis. J Hum Lact. 2017; 33(2):422-434. Data source was more recent than both Renfrew et al. 2012 and Victora et al. 2016. |
| Victora CG, Bahl R, Barros AJ, França GV, Horton S, Krasevec J, Murch S, Sankar MJ, Walker N, Rollins NC. Breastfeeding in the 21st century: epidemiology, mechanisms, and lifelong effect. Lancet 2016; 387 (10017): 475–90. | Global analysis.  Outcomes for the baby: death due to infectious disease and due to prematurity, occurring after the 1^st^ week of life  Outcomes for the mother: breast cancer  Study reports results of 28 systematic reviews and meta-analyses [22 of which were commissioned by WHO] on the association between breastfeeding and outcomes to mothers and babies |
| Walters D, Horton S, Siregar AY, Pitriyan P, Hajeebhoy N, Mathisen R, Phan LT, Rudert C. The cost of not breastfeeding in Southeast Asia. Health Policy Plan 2016; 31(8):1107-16. | Southeast Asian study.  Outcomes for the baby: cognitive outcomes, child mortality, diarrhoea and pneumonia  Outcomes for the mother: breast cancer  Data on association between breastfeeding baby outcomes specific to developing countries; data on association between breastfeeding and breast cancer obtained from Victora et al. 2016. |
| Walters DD, Phan LTH, Mathisen R. The cost of not breastfeeding: global results from a new tool. Health Policy Planning 2019; 34(6):407-417. | Global analysis  Outcomes for the baby: diarrhoea, pneumonia, mortality due to diarrhoea and pneumonia, obesity, cognitive outcomes  Outcomes for the mother: breast cancer, ovarian cancer, type 2 diabetes  Formula feeding costs  Data on association between breastfeeding and outcomes for the mother and the baby obtained from Victora et al. 2016 |

*NEC: necrotising enterocolitis*

*SIDS: sudden infant death syndrome*

### List of excluded modelling studies and reasons for exclusion

| **Excluded study full reference** | **Reason for exclusion** |
| --- | --- |
| Berridge K, Hackett AF, Abayomi J, Maxwell SM. The cost of infant feeding in Liverpool, England. Public Health Nutr. 2004; 7(8):1039-46. | Not a modelling study. Reports costs to the mother relating to feeding a baby (e.g. bottles, nursing bras etc). |
| Hansen K. Breastfeeding: a smart investment in people and in economies. Lancet 2016; 387(10017):416. | Commentary |
| Langabeer J. Applications of microcosting economic analysis in breastfeeding. Journal of Human Lactation 2018; 34(1):: 84-85 | Commentary |
| Michie C. Breastfeeding will reduce many NHS budgets. London Journal of Primary Care 2015; 7(4), 61-65. | Opinion paper. Includes references to modelling studies that have been checked. |
| Noonan MC, Rippeyoung PL. The economic costs of breastfeeding for women. Breastfeed Med 2011; 6:325-7. | Study of women's employment status and fathers' involvement associated with breastfeeding; no costs reported. |
| Phelps CE. Economics of healthcare financing: implications for breastfeeding. Breastfeed Med 2010; 5(5):191-9 | Discussion on healthcare financing in US and priorities; refers to Bartick et al. 2010 which was included in the review |
| Phelps CE. Economic issues of breastfeeding. Breastfeed Med 2011; 6:307-11. | Discussion of family /employer /insurer perspectives relating to breastfeeding |
| Santacruz-Salas E, Aranda-Reneo I, Hidalgo-Vega Á, Blanco-Rodriguez JM, Segura-Fragoso A. The Economic Influence of Breastfeeding on the Health Cost of Newborns. Journal of Human Lactation 2019; 35(2): 340-348. | Not a modelling study – observational cohort study |
| Saunders JB. The economic benefits of breastfeeding. NCSL legisbrief 2010; 18(1): 1-2 | Editorial |
| Smith JP. "Lost milk?": Counting the economic value of breast milk in gross domestic product. J Hum Lact 2013; 29(4):537-46. | Assessment of the economic value of lost breast milk |
| Smith JP, Forrester R. Who pays for the health benefits of exclusive breastfeeding? An analysis of maternal time costs. J Hum Lact 2013; 29(4):547-55. | Reports maternal time costs of exclusive breastfeeding |
| Smith JP. Counting the cost of not breastfeeding is now easier, but women's unpaid health care work remains invisible. Health Policy Planning 2019; 34(6):479-481. | Commentary on costs relating to women’s unpaid time caring for sick children |
| Walters D, Eberwein JD, Sullivan LM1, D'Alimonte MR, Shekar M. Reaching the Global Target to Increase Exclusive Breastfeeding: How Much Will It Cost and How Can We Pay for It? Breastfeed Med 2016; 11:413-5. | Assessment of the cost of investment to achieve exclusive breastfeeding targets – low and middle income countries; benefits and cost-savings from breastfeeding not modelled |
