# Supplementary material for: The cost-effectiveness of antenatal and postnatal education and support interventions for women aimed at promoting breastfeeding in the UK
Source: BMC Public Health. 2022 Jan 22;22:153. doi: 10.1186/s12889-021-12446-5 (PMC8783468; doi:10.1186/s12889-021-12446-5)
Supplement: Supplementary file 2 — Additional file 2. Evidence on clinical conditions associated with breastfeeding that was considered in the development of the economic analysis on the cost-effectiveness of breastfeeding interventions. [file 12889_2021_12446_MOESM2_ESM.docx]

**Supplementary File 2.**

**Evidence on clinical conditions associated with breastfeeding that was considered in the development of the economic analysis on the cost-effectiveness of breastfeeding interventions**

Clinical conditions that are potentially associated with breastfeeding, as considered by Renfrew et al. [1] and/or Victora et al. [2]

| Clinical condition | Modelled in Renfrew at al. [1]? | Evidence reported in Victora et al. [2] | | |
| --- | --- | --- | --- | --- |
|  |  | Conclusion on association with breastfeeding | Number of studies | Data (mean effect [95% CI]) |
| Breast cancer – mother | Yes, full | Consistent protective effect | 76  47 | Highest vs lowest duration of breastfeeding:  OR 0.81 [0.77 to 0.86]  Some evidence of publication bias and of inappropriate adjustment  for confounders  Thoroughly adjusted pooled analysis:  Ever vs. never breastfeeding:  OR 0.96 for every 12 months of breastfeeding |
| Gastrointestinal infection – baby  [Diarrhoea] | Yes, full | Strong evidence of major protection | 23  11  15  9 | More versus less breastfeeding:  Incidence: RR 0.37 [0.27 to 0.50] age <6 months  Incidence: RR 0.46 [0.28 to 0.78] age 6 months to 5years  Incidence: RR 0.69 [0.58 to 0.82] age <5 years  Hospitalisation: RR 0.28 [0.16 to 0.50] age <5 years  Most studies from low and medium income countries, where effects would be likely underestimated due to confounding  Confounder-adjusted studies showed similar effects |
| Respiratory tract infection – baby  [RTI] | Yes, full | Strong evidence of protection | 16  17 | More versus less breastfeeding:  Incidence or prevalence - lower RTI: RR 0.68 [0.60 to 0.77] age <2 years  Hospitalisation - any RTI: RR 0.43 [0.33 to 0.55] age <2 years  Most studies from low and medium income countries, where effects would be likely underestimated due to confounding  Confounder-adjusted studies showed similar effects  No evidence of publication bias |
| Acute otitis media – baby | Yes, full | Consistent evidence of protection during first 2 years of age; no evidence of protection after 2 years of age | 11  5 | More versus less breastfeeding:  OR 0.67 [0.62 to 0.72] age ≤2 years  OR 1.21 [0.60 to 2.45] age >2 years  Weak evidence of publication bias |
| Necrotising enterocolitis – baby  [neonatal units] | Yes, full | Evidence of protection | 4 | Ever vs. never breastfeeding:  58% reduction [4% to 82%]  No quality assessment by [2]; evidence considered by [1] |
| SIDS – baby | Only narrative assessment due to uncertainty around the scale of the effect | Evidence of protection | 6 | Ever vs. never breastfeeding:  36% reduction [19% to 49%]  No quality assessment by [2]; evidence considered by [1] |
| Cognitive  outcomes – baby  [Intelligence] | Only narrative assessment due to uncertainty around the scale of the effect | Consistent evidence of effect | 16  9 | Ever vs never or longer vs shorter duration of breastfeeding:  IQ increase: 3.44 [2.30 to 4.58] from childhood through adulthood  After adjusting for mother’s IQ:  IQ increase: 2.62 [1.25 to 3.98] from childhood through adulthood |
| Obesity – baby | Only narrative assessment due to uncertainty around the scale of the effect | Suggestive evidence of protection | 113 | Ever vs never or longer vs shorter duration of breastfeeding:  OR 0.74 [0.70 to 0.78] from childhood through adulthood  Some evidence of publication bias |
| Ovarian cancer – mother | No, important outcome but inadequate evidence for modelling | Suggestive evidence of protection | 41  NR | Highest vs lowest duration of breastfeeding:  OR 0.70 [0.64 to 0.75]  Some evidence of publication bias  After adjustment for parity and exclusion of nulliparous women:  OR 0.82 [0.75 to 0.89] |
| Type 2 diabetes – mother | No, limited evidence from a single study | Restricted evidence of protection | 6 | Highest vs lowest duration of breastfeeding:  OR 0.68 [0.57 to 0.82]  Adjusted for several confounding factors |
| Asthma or wheezing – baby | No, inadequate exposure and outcome measures; environmental genetic, and dietary factors interact | Inconclusive evidence of association | 29  16 | More versus less breastfeeding:  OR 0.91 [0.85 to 0.98] age 5-18 years  After thorough control for confounders:  OR 0.95 [0.85 to 1.06] |
| Diabetes – baby | No, inadequate evidence for modelling | Restricted evidence of protection | 11 | Ever vs never or longer vs shorter duration of breastfeeding:  OR 0.65 [0.49 to 0.86] childhood through adulthood  No evidence of publication bias |
| Leukaemia – baby | No, inadequate evidence for modelling | Some evidence of protection | 18 | Any breastfeeding for ≥ 6 months vs no/shorter breastfeeding:  19% reduction [11% to 27%] in childhood incidence  No quality assessment by [2]. Evidence derived from [3], but quality of the meta-analysis questioned by [4] |
| Coeliac disease – baby | No, inadequate evidence for modelling | Not assessed | | |
| Cardiovascular disease – baby | No, evidence based mainly on bio-markers rather than disease; inadequate for modelling | Not assessed | | |
| Sepsis – baby  [neonatal units] | No, inadequate exposure measures | Not assessed | | |
| Mortality due to infectious diseases – baby | Not considered for modelling | Consistent evidence of major protection | 3  3  2  9 | Exclusive vs predominant breastfeeding:  OR 0.59 [0.41 to 0.85] age <6 months  Exclusive vs partial breastfeeding:  OR 0.22 [0.14 to 0.34] age <6 months  Exclusive breastfeeding vs none:  OR 0.12 [0.04 to 0.31] age <6 months  Any breastfeeding vs none:  OR 0.48 [0.38 to 0.60] age 6-23 months  All studies from low and medium income countries, where effects would be likely underestimated due to confounding  Confounder-adjusted studies showed similar effects |
| Eczema – baby | Not considered for modelling | No evidence of association | 17  20 | More vs less breastfeeding:  OR 0.95 [0.85 to 1.07] age ≤2 years  OR 1.09 [0.99 to 1.20] age >2 years  Some evidence of publication bias |
| Food allergies – baby | Not considered for modelling | No evidence of association | 10  4 | More vs less breastfeeding:  OR 1.07 [0.90 to 1.26] age ≤5 years  OR 1.08 [0.73 to 1.26] age >5 years  High heterogeneity across studies for age ≤5 years |
| Allergic rhinitis – baby | Not considered for modelling | Possible protection up to 5 years of age; no evidence of association after 5 years of age | 5  9 | More vs less breastfeeding:  OR 0.79 [0.63 to 0.98] age ≤5 years  OR 1.05 [0.99 to 1.12] age >5 years  High heterogeneity across studies for age ≤5 years |
| Systolic blood pressure – baby  Diastolic blood pressure – baby | Not considered for modelling | No evidence of association | 43  38 | Ever vs never or longer vs shorter duration of breastfeeding:  -0.80 mm Hg [-1.17 to -0.43] childhood through adulthood  -0.24 mm Hg [-0.50 to 0.02] childhood through adulthood  Evidence of publication bias  No evidence of association on systolic blood pressure observed among larger studies |
| Total cholesterol – baby | Not considered for modelling | No evidence of association | 46 | Ever vs never or longer vs shorter duration of breastfeeding:  -0.01 mmol/L [-0.05 to 0.02]  No evidence of heterogeneity across studies |
| Osteoporosis – mother | Not considered for modelling | Insufficient evidence | 4  4 | Highest vs lowest duration of breastfeeding:  Distal radius: SMD -0.132 [-0.260 to -0.003]  Femoral neck: SMD -0.142 [-0.426 to 0.142] |
| Dental carries – baby | Not considered for modelling | Consistent evidence on detrimental effect if breastfeeding lasts >12 months | 4 | Breastfeeding >12 months vs ≤ 12 months  OR 2.69 [1.28 to 5.64] age <6 years  Publication bias likely |

More versus less breastfeeding: exclusive vs non-exclusive; predominant vs partial; partial vs none; any breastfeeding vs no breastfeeding

BMI: body mass index; CI: confidence intervals; OR: odds ratio; RR: risk ratio; RTI: respiratory tract infection; SIDS: sudden infant death syndrome; SMD: standardised mean difference

Decision on clinical conditions for inclusion in the economic analysis

| Clinical condition | Decision for inclusion of the condition economic analysis – justification |
| --- | --- |
| Breast cancer – mother | Yes – modelled in [1], clinical data updated in [2] and further, more recent data of good quality were available; data on other parameters required for modelling were available |
| Gastrointestinal infection – baby | Yes – modelled in [1], clinical data updated in [2], data on other parameters required for modelling were available |
| Respiratory tract infection – baby | Yes – modelled in [1], clinical data updated in [2], data on other parameters required for modelling were available |
| Acute otitis media – baby | Yes – modelled in [1], clinical data updated in [2], data on other parameters required for modelling were available |
| Necrotising enterocolitis – baby [neonatal units] | No – modelled in [1] but population not relevant to the analysis (babies in neonatal units) |
| SIDS – baby | Yes – only narrative assessment in [1] but possible to model; it is noted that [2] did not report up-to-date evidence on the association with breastfeeding but reported data reviewed by [1] |
| Cognitive outcomes – baby | No – economic consequences (productivity) beyond the perspective of the analysis |
| Obesity – baby | No – only narrative assessment in [1]. [2] shows suggestive evidence of association with breastfeeding. However, obesity is affected by multiple factors which may have an effect during different time periods over a person’s life and modelling obesity related exclusively to non-breastfeeding would be particularly complex and uncertain. |
| Ovarian cancer – mother | No – suggestive evidence of effect in [2] but not modelled in [1], so modelling would require identification and collection of several model parameters; moreover, low incidence of ovarian cancer meant that clinical and economic benefits per woman resulting from breastfeeding are likely to be small compared with other clinical conditions |
| Type 2 diabetes – mother | No – not modelled in [1], restricted evidence of protection in [2] and diabetes is affected by multiple factors impacting on different time periods over a person’s life, so modelling diabetes related exclusively to non-breastfeeding would be particularly complex and uncertain |
| Asthma or wheezing – baby | No – inconclusive evidence of association with breastfeeding in [2] |
| Diabetes – baby | No – not modelled in [1], restricted evidence of protection in [2] and complex modelling required |
| Leukaemia – baby | No – not modelled in [1], some evidence of protection in [2] but quality of the existing evidence has been questioned [4]. Moreover, relatively complex modelling required |
| Coeliac disease – baby | No – not modelled in [1] due to inadequate evidence and no evidence update in [2] |
| Cardiovascular disease – baby | No – not modelled in [1] due to evidence being based mainly on bio-markers rather than disease, no evidence update in [2], and complex modelling required |
| Sepsis – baby  [neonatal units] | No – population not relevant economic analysis (babies in neonatal units) |
| Mortality due to infectious diseases – baby | Yes – not considered for modelling by [1] but consistent evidence of major protection in [2] and modelling feasible; evidence came from low and medium income countries, so findings may not be directly relevant to the UK |
| Eczema – baby | No – not considered for modelling by [1] and no evidence of association found in [2] |
| Food allergies – baby | No – not considered for modelling by [1] and no evidence of association found in [2] |
| Allergic rhinitis – baby | No – not considered for modelling by [1]; only possible protection up to 5 years of age according to [2] and clinical benefits and cost-savings per person resulting from breastfeeding relatively small compared with other clinical conditions |
| Systolic and diastolic blood pressure – baby | No – not considered for modelling by [1] and no evidence of association in [2] |
| Total cholesterol – baby | No – not considered for modelling by [1] and no evidence of association in [2] |
| Osteoporosis – mother | No – not considered for modelling by [1] and insufficient evidence of association in [2] |
| Dental carries – baby | No – not considered for modelling by [1] and period of breastfeeding required to lead to dental carries is beyond the timeframe over which outcomes (breastfeeding rates) were measured in the guideline systematic review of breastfeeding interventions |

**References**

1. Renfrew MJ, Pokhrel S, Quigley M, McCormick F, Fox-Rushby J, Dodds R, et al. Preventing disease and saving resources: the potential contribution of increasing breastfeeding rates in the UK. Commissioned by UNICEF UK; 2012. <https://www.unicef.org.uk/babyfriendly/wp-content/uploads/sites/2/2012/11/Preventing_disease_saving_resources.pdf>. Accessed 14 November 2019.

2. Victora CG, Bahl R, Barros AJ, Franca GV, Horton S, Krasevec J, et al. Breastfeeding in the 21st century: epidemiology, mechanisms, and lifelong effect. Lancet. 2016;387(10017):475-490.

3. Amitay EL, Keinan-Boker L. Breastfeeding and Childhood Leukemia Incidence: A Meta-analysis and Systematic Review. JAMA Pediatr. 2015;169(6):e151025.

4. Ojha RP, Asdahl PH: Breastfeeding and Childhood Leukemia Incidence: Duplicate Data Inadvertently Included in the Meta-analysis and Consideration of Possible Confounders. JAMA Pediatr. 2015;169(11):1070.
