# Supplementary material for: The cost-effectiveness of antenatal and postnatal education and support interventions for women aimed at promoting breastfeeding in the UK
Source: BMC Public Health. 2022 Jan 22;22:153. doi: 10.1186/s12889-021-12446-5 (PMC8783468; doi:10.1186/s12889-021-12446-5)
Supplement: Supplementary file 3 — Additional file 3. Age-specific incidence of breast cancer, mortality, cost and utility data that informed the economic model component on women’s breast cancer. [file 12889_2021_12446_MOESM3_ESM.docx]

**Supplementary File 3.**

**Age-specific incidence of breast cancer, mortality, cost and utility data that informed the economic model component on women’s breast cancer**

**Age-specific incidence of breast cancer in women**

The age-specific incidence of breast cancer in women in the general population, obtained from national sources [1], is shown in Supplementary Table 1.

Supplementary Table 1. Incidence (new cases) and mortality of breast cancer in women in the general population

| **Age** | **Incidence – new breast cancer cases per 100,000 women [1]** | **Mortality due to breast cancer per 100,000 women [2]** |
| --- | --- | --- |
| 15 to 19 | 0.1 | 0 |
| 20 to 24 | 1.5 | 0.1 |
| 25 to 29 | 10.7 | 0.9 |
| 30 to 34 | 30.4 | 3.1 |
| 35 to 39 | 65.1 | 7.7 |
| 40 to 44 | 123.9 | 13.8 |
| 45 to 49 | 217.1 | 23.4 |
| 50 to 54 | 282.0 | 35.2 |
| 55 to 59 | 278.9 | 41.8 |
| 60 to 64 | 344.6 | 49.3 |
| 65 to 69 | 419.2 | 63.3 |
| 70 to 74 | 370.9 | 80.8 |
| 75 to 79 | 407.1 | 112.9 |
| 80 to 84 | 445.0 | 160.1 |
| 85 to 89 | 466.6 | 220.5 |
| 90+ | 459.4 | 317.1 |

**Mortality**

Mortality in women without breast cancer was derived from age-specific mortality data for women in the general population [3]. It is acknowledged that women in the general population include women with breast cancer, who have higher mortality than women without breast cancer, and therefore the mortality of women without breast cancer in the model has been overestimated. However, because women with breast cancer are only a very small proportion of women in the general population, the overestimation of mortality in women without breast cancer in the economic model was probably negligible.

For women with breast cancer, mortality in every model cycle was estimated using age-specific data on mortality of women in the general population [3], age-specific data on mortality due to breast cancer in women in the general population [2] (see Supplementary Table 1) and the following assumptions:

- The general population comprises women with and without breast cancer
- Women with breast cancer may die from breast cancer or from other causes
- Women without breast cancer may die from other causes only (i.e. any cause except breast cancer)
- Mortality due to other causes (any cause except breast cancer) is overall the same for women with breast cancer and those without; it is acknowledged that there is uncertainty around this assumption and that women with breast cancer may have higher or lower mortality due to other causes compared with women without breast cancer, but no relevant data were available to allow differential assumptions. On balance, we considered our assumption to be reasonable.

Mortality in women with breast cancer depends on their age but also on the number of years lived with breast cancer (duration of breast cancer). A risk ratio (RR) of mortality in women with breast cancer between 1-10 years after diagnosis versus women with breast cancer in the first year after diagnosis was estimated, using age-adjusted net survival data for women with breast cancer over 1-10 years after diagnosis [4]. Survival data and the estimated RRs are shown in Supplementary Table 2. From these data, and using (i) the estimated age-specific mortality in women with breast cancer in every model cycle and (ii) the number of women with breast cancer for 1, 2, 3 and up to 10 years after diagnosis in every cycle, it was possible to estimate the age- and breast cancer’s duration-specific mortality in women with breast cancer, depending on the number of years after diagnosis (that is, number of years lived with breast cancer).

Supplementary Table 2. Age-adjusted survival from breast cancer in women over 1-10 years from development and estimated mortality

| Year | Age-adjusted % net survival up to 10 years after diagnosis [4] | Estimated mortality in those alive at the start of each year | Estimated risk ratio of mortality in years 1 to 10 versus year 1 |
| --- | --- | --- | --- |
| 1 | 0.960 | 0.040 | 1.00 |
| 2 | 0.933 | 0.028 | 0.70 |
| 3 | 0.908 | 0.027 | 0.67 |
| 4 | 0.886 | 0.024 | 0.61 |
| 5 | 0.866 | 0.023 | 0.56 |
| 6 | 0.848 | 0.021 | 0.52 |
| 7 | 0.830 | 0.021 | 0.53 |
| 8 | 0.814 | 0.019 | 0.48 |
| 9 | 0.798 | 0.020 | 0.49 |
| 10 | 0.784 | 0.018 | 0.44 |

Women with breast cancer surviving after 10 years with breast cancer were assumed to return to the mortality of the women in the general population (rather than retaining an increased mortality associated with breast cancer for the rest of their lives), but were at risk of developing a new breast cancer (in which case their mortality increased). This assumption was necessary as no relevant UK survival data for women with breast cancer beyond 10 years after diagnosis were available in the literature and it was considered reasonable because mortality of women with breast cancer after 10 years from diagnosis is not expected to differ considerably from that of women of the same age in the general population, unless women experience a recurrence of breast cancer. Given that women were at risk of developing a new breast cancer after 10 years from initial breast cancer diagnosis, the impact of this assumption on the results is expected to be minimal.

**Healthcare costs**

Healthcare costs incurred by women with breast cancer and those without were obtained from a study that utilised data from national databases (National Cancer Data Repository, Hospital Episode Statistics, and the National Schedules of Reference Costs) on 359,771 women with breast cancer in England [5]. The study reported annual healthcare costs for each year of breast cancer between 1-9 years after diagnosis and between 1-3 years before diagnosis. Costs were reported separately for women aged 18-64 years, and those ≥ 65 years. Based on the available data, the following costs were estimated and used in our analysis:

- For women with breast cancer one year after diagnosis, the reported cost figure for one year after diagnosis was combined with the excess cost one year before diagnosis (the cost one year before diagnosis of breast cancer was notably higher than the cost incurred over 2 and 3 years before diagnosis).
- For women with breast cancer 2-9 years after diagnosis in the model, the respective reported cost figures for 2-9 years after diagnosis were used.
- For women with breast cancer 10 years after diagnosis in the model, the healthcare cost reported for 9 years after diagnosis was used, due to lack of cost data specific to 10 years after diagnosis.
- After 10 years from breast cancer diagnosis, it was assumed that women incurred the same costs as women without breast cancer, unless they developed a new breast cancer.
- For women without breast cancer, averaged costs for 3 and 2 years before diagnosis of breast cancer were used.

Depending on the women’s age in the model, relevant data for women aged 18-64 years or ≥ 65 years were used.

Reported cost data from that study [5] were updated to 2018 prices using the hospital and community health services index up to year 2014, and the consumer prices index for health for years between 2014 and 2018 [6]. Annual healthcare costs for women with and without breast cancer are shown in Supplementary Table 3.

Supplementary Table 3. Annual healthcare costs for women with breast cancer and women without breast cancer (based on [5], 2018 prices)

| Health state | Cost in women aged 18-64 years | Cost in women aged ≥ 65 years |
| --- | --- | --- |
| No breast cancer | £196 | £470 |
| Breast cancer – year 1 | £12,836 | £9,549 |
| Breast cancer – year 2 | £4,132 | £3,007 |
| Breast cancer – year 3 | £2,446 | £2,552 |
| Breast cancer – year 4 | £2,003 | £2,566 |
| Breast cancer – year 5 | £1,920 | £2,457 |
| Breast cancer – year 6 | £1,850 | £2,498 |
| Breast cancer – year 7 | £1,640 | £2,384 |
| Breast cancer – year 8 | £1,610 | £2,410 |
| Breast cancer – year 9 | £1,479 | £2,559 |
| Breast cancer – year 10 | £1,479 | £2,559 |

**Utility values**

Utility values for women with breast cancer were estimated based on data reported in a systematic review and meta-analysis of utility values for breast cancer [7]. The study reported a mean utility value for early breast cancer between 0.648 and 0.725; and for metastatic breast cancer between 0.614 and 0.640. To estimate the proportion of women with metastatic breast cancer among women with breast cancer, the following data were utilised: among prevalent cases of women with metastatic breast cancer, 28% have de novo stage IV (metastatic) disease and 72% have progressed from initially stage I-III (non-metastatic) breast cancer [8]. Between 6% and 7% of women with breast cancer have metastases at diagnosis [9]. By combining these data and assuming similar mortality over time, the proportion of women with metastatic breast cancer among women with breast cancer was estimated at approximately 23%. Given that mortality of metastatic breast cancer is higher than non-metastatic breast cancer, the proportion of breast cancer cases that are metastatic at any time was assumed to reach 20%. Using this estimate and averaging between the lowest and highest utility for early breast cancer and for metastatic breast cancer, the mean utility value for breast cancer was 0.67. This value was used for years 1-5 following diagnosis of breast cancer. For years 6-10 after breast cancer diagnosis, the mean utility value of women with breast cancer was estimated as the average between the utility of breast cancer (0.67) and the age-specific utility of women without breast cancer. After 10 years with breast cancer, women returned to the utility value of women without breast cancer (i.e. the age-specific utility of women in the general population), unless they developed a new breast cancer.

**References**

1. Cancer Resesarch UK: Breast Cancer (C50): Average Number of New Cases per Year and Age-Specific Incidence Rates per 100,000 Population, Females, UK, 2014-2016. Cancer Research UK; 2019. <https://www.cancerresearchuk.org/health-professional/cancer-statistics/statistics-by-cancer-type/breast-cancer/incidence-invasive#heading-One>. Accessed 19 November 2019.

2. Cancer Resesarch UK: Breast Cancer (C50): Average Number of Deaths Per Year and Age-Specific Mortality Rates per 100,000 Females, UK, 2014-2016. Cancer Resesarch UK; 2019. Available from: <https://www.cancerresearchuk.org/health-professional/cancer-statistics/statistics-by-cancer-type/breast-cancer/mortality>. Accessed 19 November 2019.

3. Office for National Statistics: National Life Tables, England, 2015-2017. Office for National Statistics; 2018. <https://www.ons.gov.uk/releases/nationallifetablesuk2015to2017>. Accessed 19 November 2019.

4. Cancer Resesarch UK: Breast Cancer (C50): 2010-2011 Net Survival up to Ten Years after Diagnosis, Adults (Aged 15-99), England and Wales. Cancer Resesarch UK; 2019. <https://www.cancerresearchuk.org/sites/default/files/cstream-node/surv_curve_breast.pdf>. Accessed 19 November 2019.

5. Laudicella M, Walsh B, Burns E, Smith PC. Cost of care for cancer patients in England: evidence from population-based patient-level data. Br J Cancer. 2016;114(11):1286-1292.

6. Curtis L, Burns A: Unit Costs of Health & Social Care 2018. Canterbury: PSSRU, University of Kent; 2018.

7. Peasgood T, Ward SE, Brazier J. Health-state utility values in breast cancer. Expert Rev Pharmacoecon Outcomes Res. 2010;10(5):553-566.

8. Mariotto AB, Etzioni R, Hurlbert M, Penberthy L, Mayer M. Estimation of the Number of Women Living with Metastatic Breast Cancer in the United States. Cancer Epidemiol Biomarkers Prev. 2017;26(6):809-815.

9. Cancer Resesarch UK: Breast Cancer (C50): Breast Cancer incidence by stage at diagnosis. Cancer Resesarch UK; 2014. <https://www.cancerresearchuk.org/health-professional/cancer-statistics/statistics-by-cancer-type/breast-cancer/incidence-invasive#heading-Three>. Accessed 19 November 2019.
